# Autophagy in protists and their hosts: When, how and why?

**DOI:** 10.1080/27694127.2022.2149211

**Published:** 2023-03-09

**Authors:** Patricia Silvia Romano, Takahiko Akematsu, Sébastien Besteiro, Annina Bindschedler, Vern B. Carruthers, Zeinab Chahine, Isabelle Coppens, Albert Descoteaux, Thabata Lopes Alberto Duque, Cynthia Y. He, Volker Heussler, Karine G. Le Roch, Feng-Jun Li, Juliana Perrone Bezerra de Menezes, Rubem Figueiredo Sadok Menna-Barreto, Jeremy C. Mottram, Jacqueline Schmuckli-Maurer, Boris Turk, Patricia Sampaio Tavares Veras, Betiana Nebai Salassa, María Cristina Vanrell

**Affiliations:** a Laboratorio de Biología de Trypanosoma cruzi y de la célula hospedadora. Instituto de Histología y Embriología de Mendoza. Universidad Nacional de Cuyo. (IHEM-CONICET-UNCUYO). Facultad de Ciencias Médicas. Universidad Nacional de Cuyo. Av. Libertador 80 , Mendoza, Argentina; b Department of Biosciences, College of Humanities and Sciences, Nihon University, Tokyo, Japan; c LPHI, Univ Montpellier, CNRS, Montpellier, France; d Institute of Cell Biology.University of Bern. Baltzerstr, Bern, Switzerland; e Department of Microbiology and Immunology, University of Michigan School of Medicine, Ann Arbor, MI, USA; f Department of Molecular, Cell and Systems Biology, University of California Riverside, CA, USA; g Department of Molecular Microbiology and Immunology. Department of Molecular Microbiology and Immunology. Johns Hopkins Malaria Research Institute. Johns Hopkins University Bloomberg School of Public Health. Baltimore, MD, USA; h Centre Armand-Frappier Santé Biotechnologie, Institut national de la recherche scientifique, Laval, QC, Canada; i Autophagy Inflammation and Metabolism Center, University of New Mexico Health Sciences Center, Albuquerque, NM, USA; Department of Molecular Genetics and Microbiology, University of New Mexico Health Sciences Center, Albuquerque, NM, USA; j Department of Biological Sciences, National University of Singapore, Singapore; k Laboratory of Host-Parasite Interaction and Epidemiology, Gonçalo Moniz Institute, Fiocruz-Bahia, Brazil; l Laboratório de Biologia Celular, Instituto Oswaldo Cruz, Fundação Oswaldo Cruz, Rio de Janeiro, RJ, Brazil; m York Biomedical Research Institute, Department of Biology, University of York, York, UK; n Department of Biochemistry and Molecular and Structural Biology, Jožef Stefan Institute, Ljubljana, Slovenia; o National Institute of Science and Technology of Tropical Diseases - National Council for Scientific Research and Development (CNPq), Brazil

**Keywords:** Autophagy, Protists, Life-cycle, *Leishmania sp.*, *Plasmodium sp.*, *Toxoplasma gondii*, *Trypanosoma brucei*, *Trypanosoma cruzi*

## Abstract

Pathogenic protists are a group of organisms responsible for causing a variety of human diseases including malaria, sleeping sickness, Chagas disease, leishmaniasis, and toxoplasmosis, among others. These diseases, which affect more than one billion people globally, mainly the poorest populations, are characterized by severe chronic stages and the lack of effective antiparasitic treatment. Parasitic protists display complex life-cycles and go through different cellular transformations in order to adapt to the different hosts they live in. Autophagy, a highly conserved cellular degradation process, has emerged as a key mechanism required for these differentiation processes, as well as other functions that are crucial to parasite fitness. In contrast to yeasts and mammals, protist autophagy is characterized by a modest number of conserved autophagy-related proteins (ATGs) that, even though, can drive the autophagosome formation and degradation. In addition, during their intracellular cycle, the interaction of these pathogens with the host autophagy system plays a crucial role resulting in a beneficial or harmful effect that is important for the outcome of the infection. In this review, we summarize the current state of knowledge on autophagy and other related mechanisms in pathogenic protists and their hosts. We sought to emphasize when, how, and why this process takes place, and the effects it may have on the parasitic cycle. A better understanding of the significance of autophagy for the protist life-cycle will potentially be helpful to design novel anti-parasitic strategies.

**Abbreviations:** AAs: amino acids; ATGs: autophagy-related proteins; ADCD; autophagy-dependent cell death; AMPK: 5’ adenosine monophosphate-activated protein kinase; CD40: Cluster of differentiation 40; gHBSS: Hanks’ Balanced Salt Solution; GO: gene ontology; IFN-γ: IFN-gamma; LC3: mammalian microtubule-associated protein light chain 3; LAP; LC3-associated phagocytosis; LECA: last eukaryotic common ancestor; 3-MA: 3-methyladenine; MTOR; Mechanistic target of rapamycin kinase; MDC: monodansylcadaverine; NDP52: nuclear dot protein 52; PAAR: *Plasmodium*-Associated Autophagy-Related response; PE: phosphatidylethanolamine: PCD: programmed cell death; PND: programmed nuclear death; PtdIns3K: class III phosphatidylinositol 3-kinase; PtdIns3P: phosphatidylinositol 3-phosphate; PV: parasitophorous vacuole; PVM: parasitophorous vacuole membrane; SNARE: soluble N-ethylmaleimide-sensitive-factor attachment receptor; SQSTM1/p62: sequestosome-1; TEM: transmission electron microscopy; TNF-α: tumor necrosis factor-alpha; TVN: tubovesicular network; Ub: ubiquitin; UPS: ubiquitin-proteasome system; Vps: vacuolar protein sorting.

## The life-cycle of parasitic protists

Protists are a diverse collection of organisms, mainly unicellular, distributed in the main groups of eukaryotic lineages. Many of them are free-living organisms that are important for the environment because they carry out processes like photosynthesis (plant-like algae) and waste decomposition (slime and water molds), while others (animal-like protists) are medically relevant because they cause a variety of human diseases.

Eukaryotic lineages; that constitute the eukaryotic tree of life (eToL), are currently grouped in a short number of supergroups or clades based almost exclusively on molecular phylogenies, in contrast to earlier models derived from molecular and other biological data [1,2]. Discoba (containing Kinetoplastida) and Metamonads, both belonging to the previous clade Excavate; Apicomplexa (included in the recently named TSAR group: Telonemia, Stramenopila, Alveolata and Rhizaria); and Amoebozoa lineages possess the most important species of protists causing human diseases [3] (Table 1).

**Table 1.** Classification of parasitic protists and associated diseases

Lineage	Representative Genera	Specie	Disease
Discoba (Kinetoplastida)	*Leishmania*	*Leishmania sp.*	cutaneous, mucocutaneous, and visceral leishmaniasis
	*Trypanosoma*	*Trypanosoma brucei*	African trypanosomiasis (sleeping sickness)
		*Trypanosoma cruzi*	American trypanosomiasis (Chagas disease)
Metamonads	*Giardia*	*Giardia lamblia*	diarrhea
	*Trichomonas*	*Trichomonas vaginalis*	vaginitis
TSAR (Apicomplexa)	*Plasmodium*	*Plasmodium sp.*	malaria
	*Toxoplasma*	*Toxoplasma gondii*	toxoplasmosis
Amoebazoa	*Entamoeba*	*Entamoeba histolytica*	dysentery. liver abscess.

The diseases caused by these parasitic protists range from very mild to life-threatening infections. Individuals whose defenses can control but not eliminate a parasitic infection become carriers and constitute a source of infection for others. Many protist infections that are asymptomatic or mild in normal individuals can be life-threatening in immunosuppressed patients, particularly those with acquired immune deficiency syndrome (AIDS). For instance, *Toxoplasma gondii*, a widely distributed parasite (infects up to a third of the world’s human population), usually causes a rather mild initial illness followed by a long-lasting latent infection [4]. AIDS patients, however, can develop fatal toxoplasmic encephalitis. The lack of effective vaccines, the paucity of reliable drugs, and other problems, including difficulties in vector control, have prompted the World Health Organization to target some specific diseases, collectively named Neglected Tropical Diseases (NTDs), for increased research and training. Four of these NTDs are infections caused by parasitic protists: malaria, American and African trypanosomiasis, and leishmaniasis.

Pathogenic protists display complex life-cycles alternating between several stages that differ in structure and activity. These parasitic forms develop in different types of hosts. The *Toxoplasma gondii* life-cycle occurs between members of the Felidae family (domestic cats and their relatives), which are the definitive hosts, and humans and other animals that are the intermediate hosts. *Plasmodium sp*. and trypanosomatids (*Leishmania sp., T. brucei, and T. cruzi*), on the other hand, alternate between insects and mammals, which are the vectors and the reservoirs of diseases, respectively. Parasitic forms found in insects are extracellular whereas mammalian forms have the ability to enter into host cells and establish their replicative niche there, a strategy to evade the host immune response. Extracellular parasites have developed other strategies to escape the immune system, as exemplified by the bloodstream forms of *Trypanosoma brucei*. This parasite possesses a large repertoire of genes encoding for Variant Surface Glycoproteins (VSG). During infection, this antigenic variation strategy allows evasion from the mammalian host immune system [5].

**Figure 1.**
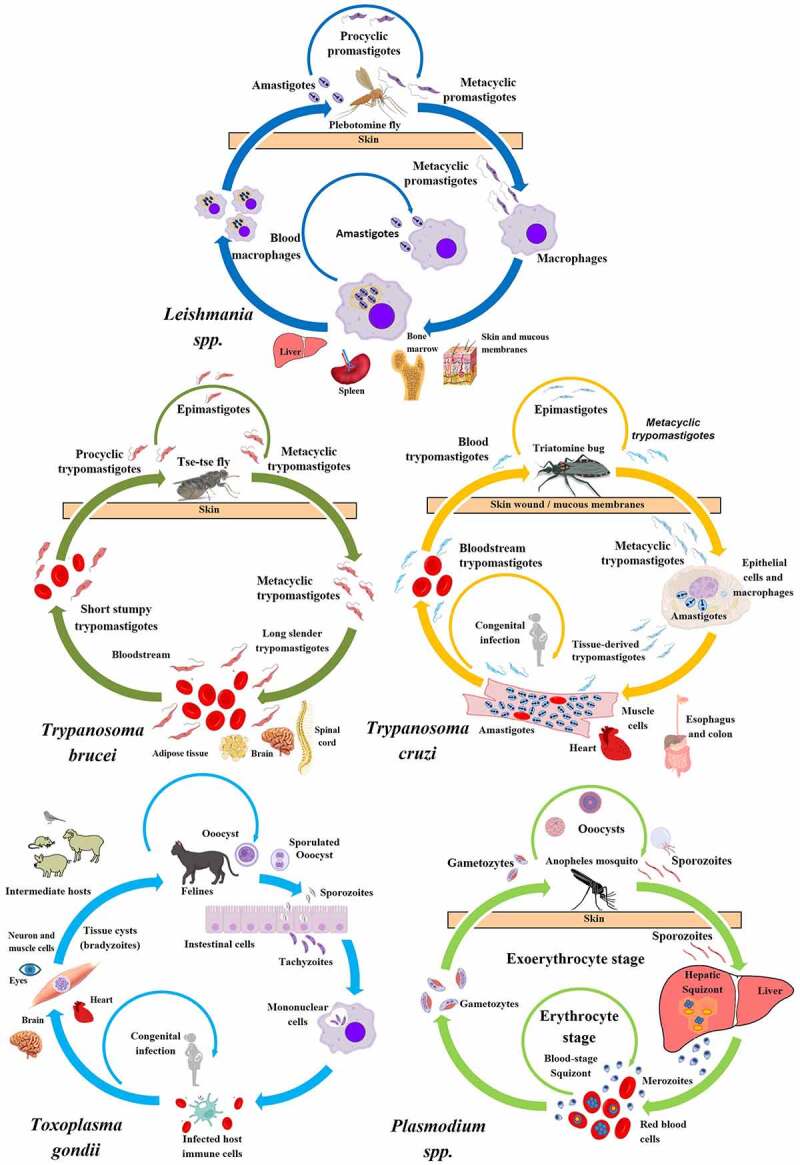
**Life-cycle of pathogenic protists**: *Leishmania spp., T. brucei, T. cruzi, T. gondii, and Plasmodium spp*. The figure shows the parasitic forms found in trypanosomatid and apicomplexan protists during their life-cycle. The main host cells targeted by these protists, the organs affected by the infections, and the vector that transmit them are depicted. Created with BioRender.com.

In the following paragraphs, we briefly present the life-cycles of protists whose connection with autophagy have been established. Figure 1 shows a graphic representation of these cycles with the developmental stages of the main parasitic protists described therein, their hosts, and the main organs they parasitize. However, it should be noted that there are other pathogenic protists including Metamonada responsible for intestinal (*Giardia lamblia*) or sexually transmitted (*Trichomonas vaginalis*) diseases, as well as pathogenic Amoebozoa (like the dysentery-causing *Entamoeba histolytica*), for which autophagy has been described to some extent, although they are much less studied.

***Leishmania*** species are transmitted by the bite of infected female phlebotomine sandflies during a blood meal. Metacyclic promastigotes, the infective stages, reach the wound and are phagocytized by macrophages and other mononuclear cells. In these cells, they differentiate in the replicative amastigote form inside a vesicular compartment, the *Leishmania* parasitophorous vacuole. Sandflies become infected by the ingestion of infected cells containing amastigotes, which transform into procyclic promastigotes in the fly gut and migrate to the proboscis as metacyclic promastigotes to start a new cycle of transmission. More than twenty *Leishmania* species can cause leishmaniasis. There are three main forms of this disease; cutaneous leishmaniasis, the most common form, occurs in the Americas, the Mediterranean basin, the Middle East and Central Asia; mucocutaneous leishmaniasis is present in Bolivia, Brazil, Peru and Ethiopia; and visceral leishmaniasis (kala-azar) is mainly found in Brazil, East-Africa and India.

***Trypanosoma brucei*** metacyclic trypomastigotes are also transmitted by the bite of an insect vector, the tsetse fly, belonging to the *Glossina* spp. Once transmitted to the blood of mammalian hosts, metacyclic trypomastigotes transform into slender trypomastigotes, with higher replicative capacity. In mammalian hosts, trypanosomes exist in three major niches: early in infection, they populate the blood; later, they breach the blood-brain barrier, and the adipose tissue constitutes a third major reservoir for *T. brucei*. Bloodstream forms of *T. brucei* multiply by binary fission in blood and other fluids and then undergo a developmental transition to the non-dividing short stumpy trypomastigotes. The cycle is completed when the tsetse fly ingests blood from an infected organism. In the fly midgut, trypomastigotes become procyclic trypomastigotes, which multiply and then leave the midgut and turn into epimastigotes. Epimastigotes migrate to the salivary glands and replicate until they transform into metacyclic trypomastigotes. Human African trypanosomiasis (sleeping sickness) is located in countries of sub-Saharan Africa. The chronic disease, produced by *T. brucei gambiense*, is the most frequent form, whereas *T. brucei rhodesiense* mainly produces acute infections.

***Trypanosoma cruzi*** is the etiologic agent of Chagas disease, a vector-borne and life-threatening illness still highly prevalent in the endemic countries of Latin America and also disseminated to non-endemic countries nowadays. The vectors of *T. cruzi* are hematophagous insects of the *Reduviidae* family, also called triatomines. After a blood meal, the infective metacyclic trypomastigotes present in the hindgut of the triatomine bug are released with the feces. Free parasites invade subcutaneous cells at the wound site and, after a short transit in a vacuole, differentiate into amastigotes, which multiply by binary fission in the host cell cytoplasm. Amastigotes then transform into trypomastigotes, which exit cells and can reach the bloodstream (blood trypomastigotes) and disseminate to different tissues. When triatomines ingest blood, trypomastigotes become epimastigotes and replicate in the midgut. In the hindgut, they transform into metacyclic trypomastigotes to restart the cycle.

***Plasmodium sp***. The causative agents of malaria are unicellular parasites of the genus *Plasmodium*, which infect different vertebrate hosts, including humans. Malaria is the most prevalent, vector-borne infectious disease worldwide. It threatens approximately half of the human population and poses a major health concern in tropical and sub-tropical regions around the globe. *Plasmodium* parasites follow a rather complex life-cycle, alternately infecting a vertebrate host and their definitive host, an *Anopheles* mosquito. A female mosquito infects a vertebrate host during a blood meal, when *Plasmodium* sporozoites are injected into the dermis. Sporozoites exhibit gliding motility that allows them to move in the skin. Sporozoites actively penetrate blood vessel walls and migrate with the bloodstream to the liver sinusoids. They invade hepatocytes and divide to form multinucleated schizonts. Transition to schizogony depends on *Plasmodium* species; always occurs in *P. falciparum* liver stage development, but does not always occur in *P. vivax*. The *P. vivax* sporozoite, once it has entered a host hepatocyte, dedifferentiates and can then become a dormant trophozoite, known as the hypnozoite. The hypnozoite can lie dormant for months and even years and then reactivate and fully develop, leading to *P. vivax* malaria relapses. Late in parasite development, merozoites egress from the liver through the budding of merosomes (merozoite-filled vesicles) to reach the sinusoidal lumen. Merosomes eventually break up inside pulmonary capillaries, resulting in merozoites liberation and red blood cell infection [6]. Within red cells, merozoites mature from ring forms to trophozoites, then to multinucleated schizonts, and finally individual merozoites (completing the intraerythrocytic cycle). Some merozoites differentiate into male or female gametocytes. These cells are ingested by the *Anopheles* mosquito and mature in the midgut, where sporozoites develop and migrate to the salivary glands of the mosquito. The mosquito completes the transmission cycle by biting another host.

***Toxoplasma gondii*** is the causal agent of toxoplasmosis, a highly disseminated infection throughout the world. The parasite primarily exists in three forms: oocysts, tachyzoites, and bradyzoites. Oocysts are only produced in the definitive host: cats and other felines. Oocysts are discharged within the feces of felines and contaminate water and produce, which may be ingested by humans and other intermediate hosts (warm-blooded vertebrates). Sporozoites developed within oocyst, are released in the intestinal lumen, infect epithelial cells and then transform into tachyzoites, which are the rapidly multiplying form of *T. gondii*. Consecutive intracellular division cycles cause tissue destruction and the spreading of the infection. Tachyzoites in pregnant women are also capable of infecting the developing fetus. Eventually, under the pressure of the host immune system, tachyzoites differentiate into tissue cysts with bradyzoites localized in muscle tissues and the central nervous system. However, in absence of a fully functional immune system, like in immunosuppressed patients or fetuses, acute toxoplasmosis arises from uncontrolled tachyzoite division. The ingestion of cysts in contaminated meat is the main source of toxoplasmosis transmission, as bradyzoites transform back into tachyzoites upon entering a new host.

Besides the classical forms of transmission described above, because of the presence of infective forms in the blood or organs of infected people, many of these parasites can also be transmitted through blood transfusion or organ transplant. Moreover, not only vertical infection from mother to child during pregnancy is important for toxoplasmosis as described above, but congenital infection is also for instance the most important form of transmission of Chagas disease nowadays and contributes to spreading the infection to non-endemic countries [7]. For Chagas disease, there is also an oral route of infection by ingestion of food and drink contaminated with the feces of triatomines [8].

## The autophagy pathway

### From yeast to mammalian cells, what we learned about autophagy over time

The autophagic process has been described morphologically in mammalian cells in the late 1950s/early 1960s, with the observation of double-membrane vesicles containing parts of the cytoplasm and organelles in various states of degradation [9]. Yet, the molecular machinery driving the biogenesis of these vesicular compartments, called autophagosomes, has remained unknown for more than 30 years. Then, in the early 1990s, using breakthrough yeast genetic screen experiments, the laboratory of Yoshinori Ohsumi identified a core set of key molecular actors in the autophagic process [10]. This set, now called autophagy-related proteins (ATGs), was then confirmed and further expanded independently by other groups using similar approaches in several yeast models and different experimental setups [11–13]. Strikingly, while there were early reports of autophagy in various animal tissues and organs, fungi, and even plants, it later appeared that the molecular machinery driving the formation of autophagosomes was largely conserved among eukaryotes [14]. In fact, perhaps because of its importance in response to nutrient limitation (which is likely a persistent challenge that many ancient or modern eukaryotes had to face at some point), autophagy is considered an early and fundamental evolutionary adaptation pathway that most likely already existed in the last eukaryotic common ancestor (LECA) [15].

Over the years the autophagic process has been extensively investigated in yeast and mammals, and to a lesser extent in model organisms like the fruit fly *Drosophila melanogaster*, the nematode *Caenorhabditis elegans*, and in some plants. However, this does not reflect the whole spectrum of eukaryotic diversity. In this regard, protists are particularly interesting as they represent dozens of major lineages, most of which share both ancestry and overall cellular characteristics with their better-studied multicellular relatives. They also provide a unique window into the diversification of modern eukaryotes [16].

Roughly 43 ATG (Autophagy-related genes) genes have been found in yeast thus far [17–19]. Although initial studies identified several homologs and orthologs across different taxa using BLAST analysis and rudimentary sequencing technology, it was not until the past decades that a clearer understanding of the different factors involved in the autophagy pathway was gained by the new molecular techniques and tools available. Using comparative genomics tools as well as genomic editing tools such as siRNA or CRISPR-Cas-9 genome editing systems, we can now piece together the progression of this system across eukaryotic evolution.

Autophagy can undergo a selective or non-selective process depending on the signaling pathways and components at play. Non-Selective Autophagy includes Macroautophagy and Microautophagy mechanisms where cytoplasmic content is randomly encapsulated and targeted for degradation in response to cellular stress [20]. Selective Autophagy targets the destruction of organelles or cellular components as a result of turnover events, or as a means to recycle damaged or redundant cell parts. Selective autophagy can target organelles such as the mitochondria (mitophagy), Peroxisomes (pexophagy), ribosomes (ribophagy), ER-specific autophagy (ERphagy or reticulophagy), and even portions of the nucleus (Piecemeal micronucleophagy, PMN). Both Macrophagy and Microphagy processes can be selective and non-selective [21,22]. Another major mechanism of the autophagic pathway that is well documented in yeast and filamentous fungi but not well understood in protist parasites is the cytoplasm-to-vacuole (Cvt) pathway. The Cvt pathway involves a series of signaling factors including hydrolases that subsequently undergoes fusion with the phagophore to form a Cvt vesicle with a double membrane. The inner membrane is then degraded, and the digestion/recycling of targeted cargo is released into the cytosol. Chaperone-mediated-autophagy (CMA) is yet another process in autophagy but has only been documented in mammalian systems [23].

While some of the players involved in the autophagic pathways have been lost, some of the core activators and co-regulators are relatively conserved and are essential among the different eukaryotic lineages.

#### The Macroautophagy Pathway

**Figure 2.**
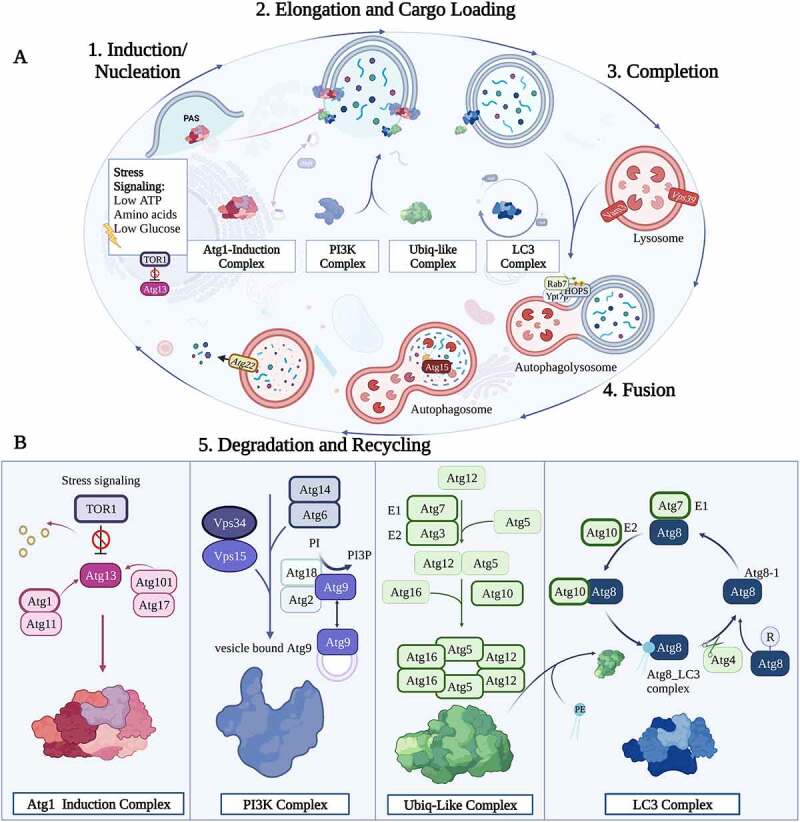
**Mechanism of Autophagy. (A)** The five steps of the autophagy pathway. 1. Induction/Nucleation begins with the recruitment of ATG induction complex factors (Pink) followed by PtdIns3K complex factors (Purple), recruited to interact with the ATG induction complex. 2. The Elongation step commences by the Ubiq-like complex formation (Green) to conjugate ATG8 proteins to PE through the LC3 Complex factors (Blue) which are recycled by the action of ATG4. This step undergoes elongation of the autophagosome. 3. Completion of autophagosome takes place with insertion of ATG8-PE to vesicle formed and final release with aid of ATG4 to deconjugate ATG8 from PE followed by the 4. Fusion of the autophagosome to the lysosome via several protein factors including Rap7, Ypt7p, and HOPS. 5. Degradation takes place after fusion, the inner membrane of the autophagolysosome is degraded by the action of ATG15 and other proteases. Autophagy is complete with the efflux of degraded proteins (basic molecules and amino acids) into the cytoplasm through the ATG22 efflux pump. **(B)** Schematic representation of the autophagy complexes and the Atg proteins that compose them.

The overall mechanism of macroautophagy begins with the formation of a phagophore. This process begins with the recruitment of several proteins in response to a range of stimuli from nutrient deprivation or apoptotic signaling. This entire process is tightly regulated and involves a plethora of protein complexes and activators working in coordination. Macroautophagy is best categorized in the yeast model and involves 5 main steps: 1) initiation/induction; 2) nucleation; 3) elongation; 4) fusion and 5) degradation and recycling, which are briefly described below (Figure 2).

##### Induction/ initiation

Under regular cellular conditions Mechanistic target of rapamycin kinase (MTOR) inhibits autophagy through phosphorylation of ATG1 (mammalian ULK1). Initiation/induction begins with a series of stress signals such as low ATP, glucose levels, or nutrient deprivation /starvation, which leads to reduced TOR1 serine/threonine kinase levels and dephosphorylation of the Atg1 complex. Dephosphorylated Atg1 dissociates from TOR1 and then initiates the autophagy induction complex. The ATG1 protein forms a complex with ATG13/FIP200 (a Focal Adhesion Kinase [FAK] family-interacting protein), ATG17, ATG101 (mammalian C12orf44) and RB1CC1 (RB1 Inducible Coiled-Coil 1 functioning in protein binding but no evidence is shown of its presence in yeast) to begin the next phase of autophagy [24–26].

##### Nucleation

Nucleation begins with the formation of the ATG1, ATG101, ATG13, and ATG17 complex. The formation of the membrane begins in peri-vacuolar membrane vesicles termed pre-autophagosomal structures (PAS). The nucleation event consists of a series of protein interactions beginning with the activation of Vps34 (PtdIns3KC3 in mammals) protein, a kinase responsible for the activation of phosphatidylinositol 3-phosphate (PtdIns3P). Several other cofactors are involved in this process including ATG6/BECN1, ATG14 (subunit of the PtdIns3KC3-complex 1 involved in protein binding), Vps15/PtdInsK3R4 (kinase), ATG9, ATG2 (a multifunctional protein involved in membrane tethering and in lipid transfer activity that was recently demonstrated in yeasts and mammals but not found in most protists) and ATG18/WIPI2 (a WD-repeat protein interacting with phosphoinositides [PIP2] binding protein). ATG9 is the only transmembrane ATG identified in both mammalian and yeast organisms. This component is localized in several membrane organelles such as the Golgi apparatus, endosomes, and several trans-Golgi network-derived vesicles called the Atg9/ATG9 vesicles. During starvation, these vesicles are recruited to the assembled ATG1 complex and mainly localize at the site of early autophagosome biogenesis.

##### Elongation

The Elongation phase of autophagy involves the recruitment of the protein components necessary for vesicle construction through ubiquitin-like conjugation systems. This process involves 3 enzyme-like players. The E1-like enzyme (ATG7) which functions as a homodimer used for activation of ATG8 (mammalian microtubule-associated protein light chain 3 [LC3]) and ATG12, the E2-like enzyme (ATG3), a key protein in the movement of LC3 towards the isolation membrane, and E3-like enzyme (formed by the ATG5-ATG12 complex). The conjugation of ATG12 to ATG5 is facilitated by ATG7 and ATG10. The E3-like enzyme complex then associates with ATG16 to form the ATG16·ATG5–ATG12 complex (ATG16 is found to be conserved in most eukaryotic organisms but is a non-essential component of the autophagy pathway in some unicellular protists). With the assembly of the ubiquitin-like conjugation systems complete, phosphatidylethanolamine (PE) is then conjugated to a glycine (Gly) residue exposed on ATG8/LC3-I (forming ATG8-PE/LC3-II) through processing by ATG4 (a cysteine protease responsible for the post-translational cleavage of the C-terminal residue of ATG8/LC3, to expose a conserved C-terminal glycine) as well as E1 and E2-like enzymes. This lipidation forms a soluble protein needed for the phagophore elongation. In yeast cells, the ATG8 facilitates tethering and hemifusion of liposomes containing ATG8-PE. Autophagosome biogenesis is complete with conjugation of ATG8, and the next phase can commence when the autophagosome undergoes fusion with the lysosome. As the autophagosome is entering completion, the ATG4 is used to deconjugate the ATG8-PE in the outer membrane, releasing ATG8 to be recycled back into the LC3-complex cycle. As discussed below, ATG8 has been a target of interest in the study of the autophagy pathway in protists. It is among the most highly conserved ATGs present in roughly all LCEA (apart from some extreme cases such as *Giardia* and *C. merolae*) yet it has been shown to be highly plastic in its roles, with evidence demonstrating roles in organelle development and evasion of host immune responses.

##### Fusion

The process of fusion to the lysosome is best understood in the yeast model system with the assembly of several protein families that are recruited to the stalk-like curvature of the autophagosome to the lysosome. The autophagosome is transferred toward the lysosome across microtubules. The process of fusion begins with the recruitment of Rab GTPase Ypt7p which functions in the recruitment of HOPS (homotypic fusion and protein sorting) proteins to membranes for fusion. The ATG8 proteins induce autophagy growth and fusion via stalk-mediated fusion events facilitated by the membrane curvature followed by the recruitment of several other protein family complexes such as soluble N-ethylmaleimide-sensitive-factor attachment receptor (SNARE) protein families that play an important role in membrane tethering and fusion of vesicles through the α-helical bundles, and Vam, required for the delivery of alpha-factor receptor-ligand complexes to the vacuole. Several other proteins such as Vam7, Vti, Ykt6, Mon1, and Ccz1 among others are needed to successfully complete the fusion mechanism forming the double membrane autolysosome and the next stage of autophagy commences [27,28].

##### Degradation and recycling

Post autophagosome fusion and completion, the inner membrane of the autophagic vesicle undergoes degradation by lysosomal enzymes. The low pH content of the lysosome along with the lysosomal hydrolases/lipases and proteases facilitate the process of degradation. In yeast, ATG15 functions as a putative lipase that is likely involved in the intravacuolar lysis of autophagic bodies (ATG15 has only been found in yeast model systems but not in mammalian or protists systems). By comparison, in mammalian cells, the lysosomal proteases (cathepsins B, D, and L) are required for the degradation of autophagosomal contents and the LC3-II of the intra-autophagosomal membrane. Finally, with the degradation of the contents into its simple monomeric units (e.g., amino acids, lipids) the contents are then subject to export into the cytosol through efflux pumps such as ATG22 found in yeast (no identifiable homolog of ATG22 has been found in mammalian cells or protists’ lineages).

#### Selective Autophagy pathways

Selective autophagy can comprise components of Macroautophagy, Microautophagy, and Cvt pathways. This process involves several of the main actors involved in the macroautophagy pathways but is mediated not by stress signals or nutrient deprivation but instead by specific proteins bound to damaged or redundant organelles/proteins labeled for degradation. Targeted mitophagy for example, is initiated in yeast cells when PINK1 (an inner membrane kinase found in the mitochondria) is transported to the outer membrane when damaged, subsequently recruiting PARK2/Parkin resulting in ubiquitination of mitochondrial substrates. Under healthy mitochondrial conditions, PINK1 accumulates to the inner membrane of the mitochondria through the action of PARL proteins.

Even proteins are subject to degradation through the targeted ubiquitination pathway. In yeast model systems, this process is established through SQSTM1/p62 (sequestosome 1), a ubiquitin- and LC3-binding protein. This protein identifies and targets either damaged, misfolded proteins, protein aggregates, or even bacteria for degradation. SQSTM1/p62 binding acts as an adaptor protein for ATG8-PE/LC3-II interaction which then initiates a form of macroautophagy-specific degradation, forming an autophagosome surrounding the targeted cargo [29].

The Cvt pathway is, yet an additional form of selective autophagy process found in yeast that requires ATP and GTP binding proteins to destroy specifically targeted material. Several Cvt pathway-specific ATG genes have been identified including ATG11, ATG19, ATG20, and ATG2 (among others) but have yet to be identified in mammal systems. The Cvt pathway is activated via four major aminopeptidases including the precursor Aminopeptidase I, (ApeI) that hydrolyzes their substrates. The Pre-Ape1 proteins oligomerize to form a homododecamer. The Ape1 complex interacts with ATG19, a peripheral membrane protein found in yeast, forming the Cvt Complex. Other cofactors are then recruited including the ATG11 and ATG9. The growing complex is transported to the PAS site where phagophore expansion begins. Phagophore expansion and maturation occur (abided by the interaction of ATG19 to ATG8-PE) surrounding the targeted cargo to form a Cvt vesicle. Once formed, the Cvt vesicles are targeted for autophagy through Vps51, Vps53, and Vps54 forming the VFT tethering complex in union with Vps45 and Q-SNARE protein family.

### Beyond a conserved core set of ATGs proteins: the autophagy machinery of protists shows losses and diversification

**Figure 3.**
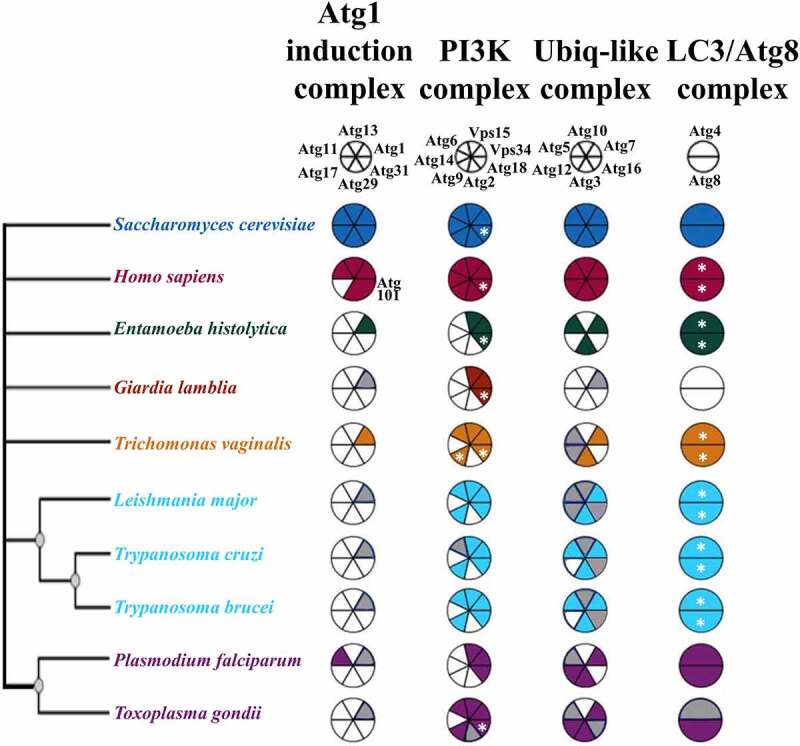
**Conservation of the core autophagy proteins in selected parasitic protists**. A Coulson plot [459] was generated to indicate the presence (color) or absence (white) of a homologous protein to the machinery described in yeast. Grey coloring indicates that only a distant homology can be found. Asterisks indicate multiple protein homologs.

The core autophagic machinery is fairly well conserved between yeasts and mammals (Figure 3). This is not surprising, as they are all part of the Opisthokonta phylogenetic group of eukaryotes and are thus relatively close from an evolutionary point of view. In the parasitic protists, database-mining studies aiming to establish the ATG repertoire in these eukaryotes highlighted some important differences with the canonical autophagy models [25,27,30,31]. Searching for homologs of yeast ATGs show there is relatively poor conservation of the early components of the machinery in parasitic protists (Figure 3). In particular, the ATG1 complex is mostly absent (the Atg1 homologs identified show significant domain variation but retain their core functional tasks). It is not currently clear whether this upstream regulating kinase complex has emerged recently in specific eukaryotic lineages, or if it has been lost secondarily in most protists; however, protists have likely evolved alternative ways of regulating the initiation of autophagosome formation.

Some key components of the membrane/lipid transfer for autophagosome biogenesis, like ATG9 or ATG2, are also seemingly absent from certain protists. The most striking reduction is in *Giardia*, a pathogen known for displaying some streamlined cellular and metabolic features that possibly only retained components whose function is important for other cellular processes, like the PtdIns3K complex. *Trypanosoma* and *Leishmania* also show the highest conservation of Vps34 and Vps15 [32] and the same for *Trichomonas* and Apicomplexa family members [33].

The conjugation systems, the Ubiquitin-like and LC3 complexes are usually quite well conserved in protists. In some species, ATG10 has been lost or has no obvious ortholog. However, a distant *Leishmania* ATG10 homolog, despite its weak sequence similarity, was shown by heterologous complementation in yeast to be likely functional [34]. Moreover, as recently shown for Apicomplexa, although ATG10 is absent and ATG12 does not have the C-terminal glycine needed for conjugation, it was shown that ATG12 could be interacting instead with ATG5 via non-covalent bonds and this way allow ATG8 lipidation [35]. These examples show that divergent protist lineages have likely evolved alternative proteins or strategies to perform functions initially described as part of the canonical pathway in yeast and mammals.

While ATG8 is present as a single protein in a few eukaryotes, ATG8 family members have expanded in others, like in the metazoan and plant lineages [36]. This is also the case in trypanosomatids, with up to 25 ATG8 homologs in *Leishmania* [37], which are categorized into four main families as *Atg8, Atg8A, Atg8B* and *Atg8C*. Atg8 and Atg8A are recruited in *L. major* during starvation and differentiation, but the Atg8A task has been shown to be reserved for puncta formation whereas Atg8 is a component of autophagosome biogenesis. Atg8B and Atg8C show no clear role in autophagy according to a 2009 study by Coombs *et al*.[37]. This resembles the situation in mammals, where several ATG8 orthologs were identified that were clustered into two subfamilies, LC3 and GATE-16/GABARAP [38]. Both subfamilies were shown to have distinct roles in autophagy with LC3B being the classical ATG8 ortholog with a major role in autophagosome formation, whereas the members of GABARAP family were more involved in the later stages of autophagy, contrary to the situation in yeast with a single ATG8 ortholog. Interestingly, the other members of the Trypanosomatidae family, *T. brucei* and *T. cruzi*, preserved three ATG8 homologs (Atg8.1, Atg8.2, and Atg8.3). Atg8.1 is proven to be the functional homolog of the yeast counterpart and remains an active component of the stress-induced autophagic pathway [39]. Atg8.2 does not seem to participate in autophagy and Atg8.3 is shown to be syntenic to that of a protein in *L. major* which exerts Atg12-like functions[40]. When a phylogenetic analysis was done comparing the protein sequences of the human members of the ATG8 family, yeast Atg8 and the *T. cruzi* and *T. brucei* Atg8.1 and Atg8.2, Atg8.1 from both *T. cruzi* and *T. brucei* clustered together with the members of the GATE/GABARAP family and yeast ATG8 that was the most distant member of the cluster with amino acid sequence similarity ~50%. Surprisingly, yeast ATG8 was found to share the lowest amino acid sequence similarity to human LC3 family members, which were grouped into another subfamily with no parasitic members, despite their common function in autophagy. Atg8.2 from *T. cruzi* and *T brucei* with no clear role in autophagy were clustered in the third subfamily (Rajkovic and Turk, unpublished). Atg12 appears to have been lost in *T. cruzi* and *T. brucei* microorganisms (although some researchers identify Atg5 and Atg10 as orthologs of Atg12) [41].

Consistent with this diversification, these parasites also express several isoforms of the ATG4 cysteine peptidase, whose function is to regulate ATG8 membrane association. It does so by processing ATG8 C-terminal extension to reveal the glycine used for lipid conjugation, and can also cut between the C-terminal carboxyl moiety and the amine group of the lipid to release ATG8 from the membrane[42]. Another unusual feature of some ATG8 homologs of trypanosomatids or Apicomplexa is that they do not have any amino acids extension after the C-terminal glycine residue, which implies ATG4 may only be used for deconjugation/delipidation in that case and suggests different modes of regulation for ATG8 membrane association [25]. While the functional implications of these particular features found in protists ATG8s are not yet fully elucidated, it is possible that this diversification reflects some differences in their physiological roles, some of which may not even be related to canonical autophagy. Interestingly, the *T. cruzi* Atg4.2, the functional analogue of ATG4, was able to process both natural Atg8 variants from *T. cruzi* and the human GATE-16, but failed to process all other human homologs (LC3B, GATE-16, GABARAP, and ATGL). In contrast, human ATG4B processed all human and parasite proteins, whereas human ATG4A processed all members of GABARAP family and Atg8.1 from *T. cruzi*, but not the others (Rajkovic and Turk, unpublished). This somehow suggests that autophagy was developing and specializing during evolution with GATE/GABARAP being perhaps more ancient, but in mammals its role was taken over by another family of proteins.

There is increasing evidence that suggests that many components of the molecular machinery for autophagy also mediates functions that are independent from canonical degradative autophagy [43]. This is for example illustrated by the implication of ATG8 and its related conjugation machinery in the inheritance during cell division of the plastid harbored by several Apicomplexa [44,45]. Hence, the apparent losses or multiplications and specific features highlighted when comparing the ATG repertoire of eukaryotes most probably reflect the functional diversification and specialization that occurred throughout evolution from the ancient function initially present in the LECA [46].

## Autophagy in the protist life-cycle

### Autophagy and protist metabolism (main physiological modulators of autophagy)

#### A brief introduction to metabolism and autophagy in eukaryotes

A conserved function of autophagy is to maintain the overall cellular homeostasis in conditions of limiting nutrients (e.g. amino acids, irons [47], or intracellular Acetyl-CoA [48]) and other metabolic perturbations (e.g., hypoxia [49], reduced energy charge [50], or increased ammonia level [51]). In these cases, autophagy enables rapid mobilization of endogenous reserves and a global rewiring of intracellular metabolism, retrieving ATP as well as building blocks to support essential biosynthetic reactions [52]. Depending on the specific type of nutrient shortage or metabolic perturbations, autophagy can be triggered via conserved or distinct metabolic sensors. The MTOR kinase is a protein complex that senses the nutrient/energy state of the cell. Inhibition of MTOR activity is strongly linked to autophagy triggered by amino acid depletion. In the presence of amino acids, MTOR is recruited and activated on the surface of the lysosomes, promoting protein synthesis by phosphorylating eukaryotic translation initiation factor 4E binding protein 1 (EIF4EBP1/4EBP1) and ribosomal protein S6 kinase (RPS6K) [53], and suppressing autophagy by phosphorylating and inhibiting ULK1, autophagy and BECN1/Beclin 1 regulator 1 (AMBRA1), ATG14 and transcription factor EB (TFEB) [54–57]. In the absence of amino acids, particularly glutamine and leucine [58], inactivated MTOR is released from the lysosomal membrane, promoting autophagy and suppressing translation. During glucose starvation, a decrease in cellular ATP boosts the activity of 5’ adenosine monophosphate-activated (AMPK), one of the key energy sensors in the cell [50,59]. As a master regulator of metabolism, AMPK stimulates autophagy by inhibiting MTOR, or by activating ULK1 or components of the PtdIns3K complex directly [54]. Importantly, activated MTOR directly downregulates AMPK signaling suggesting a bidirectional regulation between these two metabolic networks [60].

From an evolutionary perspective, autophagy is thought to be evolved from an ancient mechanism to supply nutrients to a unicellular organism encountering energy or nutrient limitations [61]. In multicellular organisms, autophagy affects not only individual cells but also the maintenance of metabolic balance in surrounding tissues or organs, further impacting the energy homeostasis of the whole body. Given the relatively simple metabolic processes and the diverse living environments, unicellular protists could be an ideal model to study the connection between autophagy and metabolism at the cellular level.

#### Amino acid transport and sensing during protist autophagy

In addition to protein synthesis, amino acids (AAs) also function as precursors for polyamines and pyrimidines biosynthesis or serve as osmolytes [62–64]. Individual eukaryotic cells can synthesize a subset of AAs, while other AAs must be uptaken from the extracellular environment. Even for AAs that can be synthesized by a cell, supplementary permeases may exist to allow uptake. The AA transporters represent one of the largest families of permeases encoded within the genomes of many eukaryotes [65]. The intracellular levels of AAs are regulated by influx from the extracellular living milieu and recycling of intracellular resources. AA sufficiency, particularly glutamine and leucine sufficiency, is sensed by two signaling pathways controlled by MTOR [66,67] and eIF2a/GCN2 [68], respectively, in higher organisms. Lacking certain AAs in the living milieu or blocking their uptake can affect global protein synthesis and induce autophagy via the inhibition of MTOR activity, through a lysosome centric “inside-out” model [69]. In the “inside-out” model, the intra-lysosomal accumulation of certain amino acids (under fed condition) induces v-ATPase-Ragulator-RagB-Raptor interaction, anchors and activates MTOR on the lysosome surface. Upon amino acids starvation, a specifically lysosome-localized, proton-coupled amino acid transporter PAT1 exports amino acids from lysosome lumen and disrupts the amino acids-sensitive Rag-Ragulator complex, releasing deactivated MTOR from the lysosome membrane [69].

Approximately 30 to 40 genes encoding putative AA permeases/transporters can be identified in each of the *L. major, T. brucei* and *T. cruzi* genomes [70]. Some of these transporters have been characterized [71] though most have not. AA-starvation triggers autophagy in many parasitic protists [72–74]. However the AA sensing mechanism remains largely unknown.

**Figure 4.**
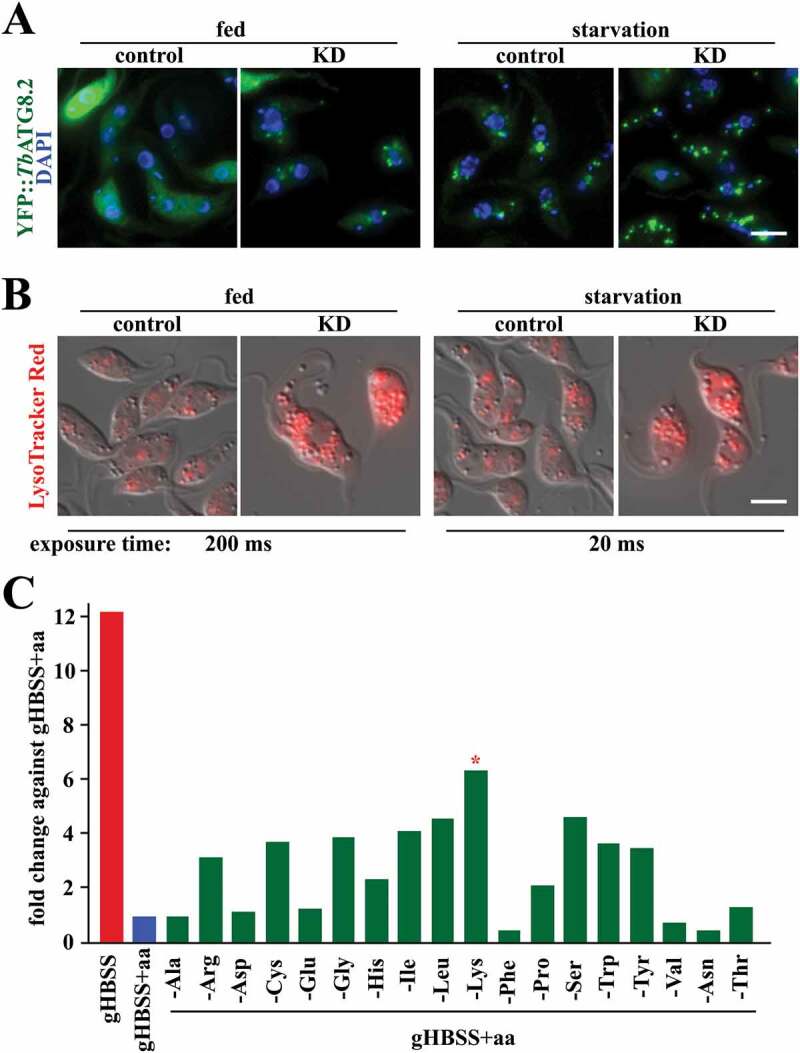
**Lysine depletion triggers autophagy in *T. brucei*. (A)** The images show the formation of autophagosomes using ectopically expressed YFP::TbATG8.2 as an autophagosome marker in parasites upon RNAi of lysine transporter TbAAT16. **(B)** Images show acidocalcisome acidification (based on the accumulation of LysoTracker Red) in parasites after RNAi of lysine transporter TbAAT16. Upon RNAi of lysine transporter TbAAT16, autophagosome formation and acidocalcisome acidification were observed in fed conditions (A and B, left panels), and these activities increased further upon starvation (A and B, right panels). **(C)** Cells ectopically expressing YFP::TbATG8.2 were incubated in gHBSS containing all AAs except for the indicated individual AA for 2 h. The autophagosome number was quantified based on YFP::TbATG8.2-positive puncta. The relative autophagy activity (fold change) was normalized against the negative control (gHBSS with full AAs), where few autophagosomes were formed. gHBSS only was used as a positive control.

Taking advantage of the inheritable and inducible RNAi system that is available in *T. brucei*, it has been performed an RNAi screening of putative AA transporters identified in the *T. brucei* genome. Upon the depletion of each individual or a group of AA transporters that share near identical DNA sequences, cell viability and autophagy were monitored. While some putative AA transporters are essential for procyclic cell growth in culture, only one transporter (encoded by Tb927.11.15840/Tb927.11.15860) had effects on both cell viability and autophagy. This transporter, named TbAAT16 [75], is a homolog of LdAAT7 and TcAAT7 in *L. donovani* and *T. cruzi*, respectively, which have characterized roles in lysine uptake[76]. The specific lysine uptake activity of TbAAT16 was also observed in *T. brucei* [75]. Upon RNAi silencing of *TbAAT16* transcripts, autophagosome number increased in both fed and starvation conditions (Figure 4A). Acidification of the acidocalcisomes (lysosome-related organelles), which has been shown to be required for autophagy induction [21], was also observed (Figure 4B), consistent with autophagy induction in *TbAAT16*-RNAi cells in both fed and starvation conditions.

The effect of individual AAs in autophagy induction was also monitored by incubating cells in the gHBSS (Hanks’ Balanced Salt Solution), an amino acid-free buffer that robustly induces autophagy in *T. brucei*, supplemented with all AAs except for the specified individual AA (Figure 4C). Individual deprivation of several amino acids led to increased autophagy, but deprivation of lysine displayed the highest autophagy level (Figure 4C). Together with the RNAi screening of the AA-transporters, these results suggested that deficiency in lysine or lysine uptake is an efficient trigger for autophagy in procyclic *T. brucei.*

In a previous study, supplementation of histidine to the gHBSS starvation buffer inhibits autophagosome formation, suggesting that the presence of histidine has an inhibitory role on autophagy [77]. Notably, histidine and lysine are two of three positively-charged AAs (the other one is arginine) stored in the lysosome-related acidocalcisomes [78]. The acidification of acidocalcisomes upon starvation is shown to be crucial for autophagy initiation [21]. Whether the lysine transporter TbAAT16 functions in lysine storage or release in/from the acidocalcisomes and whether the storage/release processes are coupled to proton transport remain unknown. However, TbAAT16 depletion led to acidocalcisome acidification both in fed and starvation conditions (Figure 4B), implying that in *T. brucei* amino acid sensing may occur via a similar “inside-out” mechanism as shown in mammalian cells [69].

#### Energy metabolism and autophagy in protists

Glucose and related hexoses, such as fructose, are important carbon sources for many types of cells. While glycolysis using glucose serves as a main ATP production pathway used by parasitic protists [79], ATP can also be produced from amino acids or fatty acids depending on their availability in the different living milieu. In mammal hosts, *T. brucei* exists in different niches. The bloodstream form (BSF) of *T. brucei* lives extracellularly in blood, a glucose-rich environment, and as a result, they metabolize high levels of glucose by glycolysis and do not possess a fully functional Krebs cycle or oxidative phosphorylation [80]. In contrast, the adipose tissue forms (ATF) utilize the exogenous fatty acids, such as myristate, to produce ATP by b-oxidation [81]. In the procyclic form (PCF) that lives in the midgut of the tsetse fly, the parasite mitochondrion is fully developed and ATP is generated by oxidative phosphorylation using proline, which is abundant in the tsetse vector and can be converted into glutamate and a-ketoglutarate prior to entering the TCA cycle [82]. *Leishmania* promastigotes in culture can use either glucose or proline, which are also important nutrients available in the sand fly [83]. When the promastigote enters a macrophage and is internalized into the phagolysosome, energy metabolism shifts to fatty acids and amino acids [84–86]. The proline-glutamate pathway is also conserved in *T. cruzi* [87]. While *T. brucei* is auxotrophic for proline [88], *T. cruzi* is capable of producing proline in its cytosol from glutamate [71]. Different to *T. brucei* and *Leishmania sp., T. cruzi* can also convert histidine to glutamate via a four-enzymatic-steps pathway [89].

In mammals, energy shortage usually occurs upon glucose starvation. AMPK senses low energy levels (i.e. high AMP: ATP ratio) and mediates autophagy initiation [54]. In protists, although glucose-starvation/restriction conditions can trigger autophagy in certain parasites such as *Trichomonas vaginalis* [90–92], the link between AMPK and autophagy is only experimentally confirmed in *D. discoideum* (see below) [93–95]. In *Plasmodium berghei*, AMPK homolog PbKIN functions in sporozoite development during the egression to the mosquito’s salivary gland [96], while the *P. falciparum* homolog PfKIN is involved in transmission from human bloodstream to mosquito midgut [97]. In *T. gondii*, the AMPKa homolog TOXPK1 responds to glycogen biosynthesis during tachyzoites to bradyzoites differentiation [98]. Similar to *Plasmodium, T. brucei* AMPK is also involved in cell differentiation from proliferative long slender form to quiescent short stumpy form [99]. Expression of a constitutively active TbAMPKa1 is sufficient to induce differentiation, and inhibiting TbAMPKa1 activity reduces differentiation in mice [99]. Interestingly, depleting TbMCU, a mitochondrial calcium uniporter in *T. brucei*, induces autophagy accompanied by an increase in cellular AMP: ATP ratio [100]. However, there is no evidence that these two events are directly connected. On the contrary, several lines of evidence suggest that TbAMPK is not involved in autophagy regulation in *T. brucei*. 1. Glucose starvation rapidly depletes cellular ATP but does not induce autophagy in either procyclic or bloodstream-form cells [101]. 2. Depletion of TbAMPK b or g subunit does not affect amino acids-starvation induced autophagy [101]. 3. TbAMPKa1 activation inhibits TbTOR4 that functions in differentiation [102], but not TbTOR1 that is proposed to function in autophagy regulation [103]. 4. Efficient autophagy induction requires a high level of cellular ATP and autophagic activity is positively correlated with cellular ATP [100]. Last but not least, glucose and proline, the two main nutrients for ATP production in the bloodstream and procyclic trypanosomes respectively, are present at a relatively constant level in the hosts [104,105]. In fact, a systematic study using multiple mammalian cell lines also found that only AA-, but not glucose-depletion induces autophagy [106]. Although glucose starvation activates AMPK, the activation does not significantly increase autophagosome formation [106]. It appears that AMPK is not an absolute requirement for autophagy induction as once thought [107].

### Autophagy in organelle turnover and protein degradation (role of autophagy in the protist differentiation)

The most striking feature of parasitic protists is their capacity of surviving in multiple vertebrate and arthropod host environments. To this end, they have evolved complex cellular cycles that entail rigorous morphological changes and circulation between vastly different hosts. These extreme shifts in external conditions throughout the life-cycle have not only imprinted on these obligate parasites the need to undergo vast structural changes but also imply extreme plasticity in their cellular processes and pathways to adjust to these ever-changing circumstances. The mechanism of autophagy lends itself as an example to the adaptive changes undergone in response to organismal adaptive pressures throughout its evolution. This process has evolved and adapted to contribute to diverse cellular functions between different protist lineages including, but not limited to, cell differentiation, protein turnover and the maintenance of the apicoplast (a non-photosynthetic plastid found in several Apicomplexa). By studying this system in parasitic human pathogens, our understanding of the autophagic process has unveiled how these protists not only use the autophagy machinery in a vastly simplified and concise manner to undergo the basic cellular recycling and organelle/protein turnover, but also rely on its components for their development and survival. In the following subsections we will present the different roles of parasite autophagy proteins (or ATGs) in the life-cycle of each pathogenic protist, from the rudimentary process found in *Entamoeba* or *Giardia* up to the more developed autophagy of trypanosomatids and Apicomplexa.

#### Entamoeba sp.

Amoeba are widespread and usually harmless, like the social amoeba *Dictyostelium discoideum*, but there are pathogenic species like *E. histolytica* (responsible for amoebic dysentery and amoebic liver abscess), and opportunistic pathogens like *Acanthamoeba* spp. (that can occasionally cause encephalitis or keratitis in humans). *D. discoideum* has been used extensively as a model for studying autophagy in amoeba [93,108]. In *D. discoideum*, MTOR, AMPK and ULK1/ATG1 are conserved with the mammalian orthologs, and their involvement in autophagy is experimentally confirmed. DdAMPKa directly interacts with ULK1/ATG1 via its N-terminal region and promotes the basal autophagy induction [109]. It also displays a fairly well conserved ATG machinery [110] and has been shown to rely on autophagy for differentiation and for its developmental program [111]. In contrast, there is reduced autophagy-related machinery in *E. histolytica* (Figure 3), which possesses a rather well-conserved ATG8 conjugation system, but for instance seems to lack the ATG1 complex that is present in *D. discoideum*. However it should be kept in mind that these two species are quite phylogenetically distant [112]. Moreover, *E. histolytica* has emerged as a parasite from a mostly nonparasitic clade. Evolutionary history and differences in the way of life may thus have shaped losses and gains within amoeba species. While common autophagy inducers such as nutrient starvation do not lead to autophagosome formation in *Entamoaeba*, autophagosome-like structures have been observed during encystation of *E. invadens* [113]. Interestingly, disrupting the autophagy in *A. castellanii* leads to a reduced cyst formation [114,115]. Altogether, this suggests autophagy could be involved in encystation of pathogenic amoeba, but clearly information obtained so far on the autophagic function is very thin and these models deserve to be studied further.

#### Giardia sp.

This parasite is seen as an enigmatic, double nucleated eukaryotic microorganism, exhibiting uniquely traits and a relatively small genome. Despite its enveloped nucleus and compartmentalized organelles, *Giardia* displays traits that are core to all eukaryotes and was believed to be asexual, although recent evidence proves the contrary. It shows no evidence of a fully functional mitochondrion, instead it retains simpler mitochondrial-like remnants called mitosomes and undergoes anaerobic metabolism. It also seems to lack classical lysosomes [116]. Consequently, the autophagy systems in *Giardia* are not well understood and even suggested to be completely absent in the pathogen. Of the 40 known ATGs across the LECA, there is very little evidence of preserved homologs found to date [117]. Until recently, Vps34 and Vps15 (which also play non-autophagic mechanisms) were thought to be the only ATGs conserved in the *Giardia* genome. However, there are claims of homology in some ATGs suggesting the existence of a primitive or incomplete form of autophagy at play [74]. In an earlier study aiming to decipher the programmed cell death (PCD) of *Giardia*, researchers were able to induce stress responses through oxidative stress (H_2_O_2_) and incubation with drugs like metronidazole. Through monodansylcadaverine (MDC) staining (an autofluorescent compound labeling autophagic vacuoles) on starved cells, the authors identified the hallmarks of an autophagy-like response. Researchers also performed genome sequencing analysis to identify potential homologs to PCD and autophagy. BLASTP queries compared amino acid and protein sequences to identify hits with high similarity index revealed homologs to some core ATGs such as TOR, ATG1 and ATG16 [73]. However, this remains to be a topic of debate and these genes have yet to be functionally characterized in later studies. In a subsequent study aimed to decipher the pathways of *Giardia* encystation and the role that MLF (Myeloid leukemia factor protein, an important regulator of cell differentiation)-dependent vesicle formation plays in this mechanism, it was found that ATG8-like proteins (Atg8L) along with a FYVE-domain containing protein (autophagy-linked protein ALFY) colocalized with the MLFVs to form a complex resulting in protein clearance. In this study the MLF, FYVE-HA and Atg8L proteins were detected in exosomes using anti-MLF and anti-HA antibodies through immunofluorescent imaging. It was later discovered that MLFVs belonged to the degradative pathway when analyzed in KO CDK2m3 cell lines. MLF, FYVE, and ATG8L were found to play a positive role in encystation and function in protein clearance pathway giving insights on protein turnover in *Giardia* [74]. A recent study, however, argues against these claims. This study attempted to identify enzymes involved in the Ub and Ub-Ls conjugation processes using the hidden Markov models (HMMs) from functional *Pfam* domains [114]. Here, researchers found 118 sequences in the *Giardia* proteome that were classified into five groups: Ub and Ub-like, E1 and E1-like, E2, E3, and DUB enzymes in *G. intestinalis*. However, they were unable to find any sequence homology to ATG8 and ATG12 proteins. They did, however, identify an ubiquitin-related protein HUB1 homolog, but it is more closely related to a human from an evolutionary point of view. The study did recognize that a novel non canonical ubiquitin-conjugating enzyme NCUBE (GL50803_8638) is localized in the endoplasmic reticulum (ER) lumen and participates in ER-associated degradation. It is yet still unclear whether or not canonical autophagy occurs in *Giardia*. Yet, as it contains minimal components needed for complex cellular processes and expresses rudimentary mechanisms, this can reflect its basal evolutionary position or genomic reduction in response to the obligate-parasite’s adaptive needs, and there might be lineage-specific innovations in this organism. This calls for further investigations as the *Giardia* parasite stands out among most other protist superfamilies because it can potentially bridge the gap of knowledge between the prokaryotic and eukaryotic lineages.

#### Trichomonads

*Trichomonads* are a family of anaerobic protists with a drastically modified autophagic repertoire. *Trichomonads* can be parasitic or thrive in host guts as commensals. Among the trichomonads of particular interest are *T. foetus* and *T. vaginalis*, which are flagellated parasites of the urogenital tract of cattle and humans, respectively. A unique trait among these microbes is their lack of mitochondrial organelles. Instead, these organisms meet their energy requirements through hydrogenosomes. Hydrogenosomes are electron-dense organelles, encapsulating enzymes that participate in the metabolism of pyruvate formed during glycolysis. *T. vaginalis* is one of the most widespread non-viral sexually transmitted infection with approximately 276 million cases reported annually worldwide [72]. Recent bioinformatics analysis showed conservation of the core TvAtg8 genes but most of the core system components of autophagy such as the Initiation complex proteins and Ub-like systems (ATG5-ATG12) were lost or modified [118]. The autophagy system of this organism was studied by *Hernandez-Garcıa et al*. in a 2018 publication in which indirect immunofluorescence assays were performed with an anti-rTvAtg8 antibody on iron- or glucose-reduced, or rapamycin treated cells. Under these stress conditions parasites presented autophagosome-like vesicles. These TvAtg8-positive vesicles were found in the highest percentage numbers upon glucose reduction conditions compared to the rapamycin or iron reduction conditions. Furthermore, sequence analysis revealed the presence of two ATG8 proteins, Atg8a which showed greater sequence identity to LC3/ATG8 while the second Atg8b more closely related to the GABARAP protein families. Regardless of the sequence similarities, both proteins were found to participate in the biogenesis of autophagosomes [119]. These findings were later expanded on by the same researchers where *T. vaginalis* was revealed to potentially undergo two different autophagic pathways depending on the stress signals initiated. This study validated the non-canonical autophagic system induced via proteasome inhibition (lactacystin and gliotoxin) or glucose reduction. Yet the ubiquitin-proteasome system (UPS) was shown to be involved in an alternative to degradative autophagy during the stationary phase of high glucose medium cultures, as proteasome inhibition initiated the formation of circular membrane whorls containing organelle fragments. This suggests that *Trichomonas* not only take part in canonical autophagic processes during instances of environmental stress but also perform basal level autophagy for proteolysis under nutrient-replete conditions [92].

#### Leishmania sp.

The repertoire of autophagy-related genes is reduced in *Leishmania* compared to yeast and human, likely a result of the drastic adaptive changes throughout the ancestral lineages of parasitic protists. No genes involved in the initiation of macroautophagy have been identified in *Leishmania*, except for a possible ATG1 ortholog, which shows significant domain variation but may retain its core function. Genes involved in the process of membrane tethering have been identified bioinformatically, including *ATG9* (seeds), *VPS34, VPS15, ATG6, ATG18* (PtdIns3K complex) and *ATG3, ATG4, ATG5 ATG7, ATG8, ATG10, ATG12* and *ATG16* (Figure 3) [25,34]. *Leishmania* Atg8 has been used as a protein marker for autophagosome formation [120] and functional characterization of all the proteins involved in the ubiquitin-like conjugation, except for ATG16, has been documented. *Leishmania ATG5, ATG10* and *ATG12* homologs were found to complement their respective *S. cerevisiae* mutants [121], whilst a functional ATG12-ATG5 conjugation system was shown to be required for ATG8-dependent autophagosome formation [122]. ATG12 is unusual in that it requires C-terminal processing by a peptidase, as yet unidentified. *Leishmania* mutants lacking ATG5 (Δ*atg5*) were unable to form autophagosomes, had a reduced flagellum and had reduced virulence, likely because of disruption of mitochondrial homeostasis [122]. *Leishmania* has two ATG4 cysteine peptidases, ATG4.1 and ATG4.2, with differing roles in processing ATG8. Individual ATG4 gene deletions could be generated (Δ*at*g*4.1* and Δ*at*g*4.2*), but not double mutants indicating that ATG4 is essential for parasite viability. There is a level of functional redundancy between the two ATG4 enzymes; ATG4.2 appears to be more important, as Δ*at*g*4.2* mutants were less virulent than wild type parasites [123] as they are defective in metacyclogenesis [120]. Interestingly, autophagy and differentiation were also affected in VPS4 mutants. VPS4 is an ATPase involved in the formation of late endosomes and VPS4 mutants were found to be defective in the fusion of autophagosomes to the endosomal/multivesicular tubule-lysosomal compartment [120]. This demonstrates the importance of the function of late endosomes and autophagy in the transformation to the infective metacyclic promastigote. Additionally, lysosomal cysteine peptidases CPA and CPB are important for autophagy, as *Leishmania* mutants deficient in the peptidases do not effectively degrade autophagosomes and fail to differentiate [34]. Short term starvation or entry into the stationary phase is effective in inducing autophagy. Such quiescent cells have a global reduction in transcription, however, a subset of transcripts did not follow this trend and were relatively upregulated in quiescent populations, including those involved in the autophagy pathway [124]. Not all mutants are defective in autophagy, indeed mutants deficient in the *L. major* PAS domain-containing phosphoglycerate kinase have an increase in autophagosome formation and cell death [125].

While little is known about the payload of autophagosomes in *Leishmania*, glycosomes, peroxisome-like organelles that uniquely compartmentalize glycolytic and other metabolic enzymes in *Leishmania*, have been identified as autophagosome cargo [126]. *L. major* Δ*atg5* mutants had significantly greater glycosome numbers in promastigotes and amastigotes, linking autophagy to glycosome homeostasis. Glycosomes were found to be cargo in ~15% of autophagosomes, which were trafficked to the lysosome for degradation. During differentiation from metacyclic promastigote to amastigote the percentage of glycosome-containing autophagosomes remained constant, yet the number of autophagosomes increased 10-fold, indicating that increased turnover of glycosomes was due to an increase in autophagy flux. Mitophagy of the single mitochondrion found in *Leishmania* was not seen in *L. major* during growth or differentiation; however, remnants of the mitochondrion formed because of stress-induced fragmentation were found in autophagosomes and lysosomes, indicating that these damaged organelles are recycled by autophagy [126].

#### T. brucei

In most eukaryotes, a conserved function of autophagy is the maintenance of cellular energy and AA homeostasis in nutrient-limiting conditions. In parasitic protist, the mutual dependence between autophagy and energy metabolism is best characterized in *T. brucei* [101], It has been well documented that *T. brucei* participates in complex morphological changes while cycling between its mammalian and the arthropods tsetse fly hosts. This change of hosts requires rapid and drastic cellular remodeling events for the parasite to adapt and thrive between the glucose abundant bloodstreams of their vertebrate hosts to the tsetse fly midgut where conditions may not provide a steady supply of glucose and other necessary nutrients. To adapt to these cycles, the parasite found in vertebrates have to shift from undergoing glycolysis as its major ATP source, carried on the peroxisome-like organelles (glycosomes), to the mitochondrial tricarboxylic acid cycle (TCA) occurred in its procyclic stages within the invertebrate hosts. Here, it develops the mitochondria expressing TCA cycle enzymes, respiratory chain, and oxidative phosphorylation enzymes to ensure its energy requirements are met [127]. The autophagy systems play an important role in the cellular remodeling of its enzymes and organelles during these host cell adaptations. These findings were a result of immunofluorescence assays performed on *T. brucei* cells using anti-aldolase (ALD) antiserum and an anti-p67 monoclonal antibody targeting glycosomes and lysosomes, (respectively). Observations showed significant increase followed by rapid decrease in glycosomal numbers in the LS (long, slender cells) and I2 (mixture of intermediary and short-stumpy trypanosomes) stages of development. These glycosomes were also seen to be colocalized and enter lysosomes membrane compartment during the differentiation processes from slender to stumpy and from stumpy to procyclic forms of the parasite’s development, expressing the importance of the autophagy system in the parasite’s morphological transitions [128]. *T. brucei* possesses four MTOR homologs (TbTOR1-4) and at least three MTOR complexes: TbTORC1, TbTORC2 and TbTORC4 [102,129–131]. While TbTORC1 and TbTORC2, respectively, maintains conserved functions in autophagy[129], and protein synthesis and cytokinesis regulation[132], TbTORC4 is unexpectedly connected with the AMPKa1 pathway and functions in *T. brucei* differentiation from the proliferative slender form to the quiescent stumpy form [99,102]. RNAi depletion of each of the four *T. brucei* mTOR homologs leads to a mild increase in autophagosomes [133]. However, due to the lack of functional ULK1/ATG1 homologs [134], the mechanism of TbTORs, particularly TbTORC1 in autophagy initiation remains unclear. The degradative activity of autophagy in this parasite also remains poorly understood. To this date, not a single parasite protein has been identified as an autophagic substrate, making autophagosome counting the only method to measure and compare autophagy activity under different conditions. However, a role of autophagy in maintaining new protein synthesis and energy production in starved cells was documented. By using the Click-iT® technique [135–137] it was demonstrated that newly synthesized proteins were significantly reduced in cells starved in gHBSS [39], compared to cells cultivated in fed conditions, A total of 372 proteins were identified as newly synthesized proteins in gHBSS-starved cells, including proteins with unknown functions (28%), and proteins involved in metabolism (24%), translation (13%), proteolysis (8%), protein folding (6%), protein transport (4%), RNA processing (4%) and other pathways (13%). Gene Ontology (GO) enrichment analysis showed that the most enriched GO terms (ranked by Benjamini adjusted p-value) were metabolism, catabolism, translation and protein degradation. KEGG enrichment analysis indicated that the two most enriched pathways were TCA cycle and glycolysis, both involved in cellular energy production. Protein expression in starved WT and autophagy-deficient TbATG8.2^−/−^ cells was also compared. Approximately 2/3 of newly synthesized proteins were found more abundantly expressed in wild type cells, and many of these proteins are involved in ATP production through glucose or proline metabolic pathways. Reduced cellular ATP level was also found in TbATG8.2^−/−^cells compared with wild type control. Altogether, these results strongly support a role of autophagy in maintaining new protein synthesis and energy production in starved cells, which further facilitates the autophagy process.

#### T. cruzi

Initial BLAST studies in the genome of trypanosomatids confidently identified genes of PtdIns3K complex and ATG8/LC3 complex [30,127] (Figure 3). These studies did not find *T. cruzi* homologs of yeast ATG2, ATG14, and ATG12 proteins. Later individual BLAST searches, found others putative *T. cruzi* versions of autophagy genes such as ATG5, ATG10, and ATG16 (with low overall homology, but with conserved specific motifs or domains, accession numbers TcCLB.509053.80, TcCLB.509965.280 for ATG5, TcCLB.509911.110 for ATG10 and Tc00.1047053506775.160, Tc00.1047053511167.20 for ATG16, H. Sakamoto, personal communication). A distant *T. cruzi* ATG1 homolog was also found but its function was still unknown. Despite the low percentage of identity, further experimental data confirmed the participation of several of these genes in *T. cruzi* autophagy. In 2008, Alvarez and coworkers cloned two ATG8 proteins from *T. cruzi*, TcAtg8.1, and TcAtg8.2, and also the autophagins TcAtg4.1 and TcAtg4.2 [41]. The authors demonstrated that autophagins have the ability to process the two homologs of ATG8 near the carboxyl terminus, exposing a conserved glycine residue and that both proteins were able to substitute the yeast homologs in functional assays. Later studies showed that TcAtg4.2 has greater proteolytic activity and it is responsible for TcAtg8.1 processing [138]. Other components of the *T. cruzi* autophagy have also been cloned and characterized. TcVps34 produces PtdIns3P and participates in osmoregulation, acidification, and vesicular trafficking [139]. TcVps15 interacts with TcVps34 to form the PtdIns3K complex that associates with cell membranes. TcVps15 has been shown to be a key regulator of TcVps34 enzymatic activity; both proteins change their subcellular distribution showing a partial colocalization with TcAtg8.1 in autophagosomes under conditions of nutritional stress [140].

Recent studies have shown the signaling pathways that allow *T. cruzi* to adapt to different stress situations. AMPK, the serine/threonine kinase activated by environmental stress with decreased ATP and increased AMP, has been identified and characterized in this parasite. This enzyme was proposed as a new regulator of nutritional stress in epimastigotes [141]. The presence of a putative *T. cruzi* MTOR gene was also suggested [142] and further confirmed with the use of rapamycin, that induces autophagy due to MTOR inhibition, similar to mammalian cells [143]. The inositol 1,4,5-triphosphate receptor (TcIP3R) was found in the acidocalcisome, a specialized compartment found in trypanosomatids characterized by acidic pH and large content of Ca^2+^ and polyphosphates. TcIP3R has been shown to be an IP3-controlled Ca^2+^ release channel required for Ca^2+^ uptake by *T. cruzi* mitochondria, regulating pyruvate dehydrogenase dephosphorylation, mitochondrial O2 consumption, and preventing autophagy [144]. Interestingly, this receptor is localized in the endoplasmic reticulum in animal cells, the main place where the autophagosome formation is initiated. As mentioned above, acidification of acidocalcisomes in *T. brucei* is a key process that precedes autophagosome formation [145]. In a similar way, inhibition of vacuolar H^+^-ATPase by bafilomycin prevents acidification of acidocalcisomes and inhibits autophagy in *T. cruzi* [146], thus evidencing a possible role of acidocalcisomes in the origin of autophagic compartments in this parasite.

The autophagy process is important for parasite survival under conditions of nutritional stress and differentiation [147]. As mentioned in the introduction, *T. cruzi* displays three different parasitic stages throughout its life-cycle and undergoes different stress conditions. One of the most studied differentiation processes is the metacyclogenesis that occurs in the gut of the insect vector when epimastigotes differentiate into metacyclic trypomastigotes. Autophagy is triggered during spontaneous metacyclogenesis of epimastigotes, which spend long periods of starvation after reaching the stationary phase of growth *in vitro*. In these experiments, the TcAtg8.1 was found in autophagosomes, only in the intermediate stages, showing that this process is very dynamic [41]. In agreement with this, an increase in the gene expression of TcAtg7 and TcAtg8 was observed during the process of metacyclogenesis [148]. Other stress conditions such as exposition to alkaline or acid media, also induce the autophagic pathway in *T. cruzi*, contributing to the differentiation process, prior to intense mitochondrial dysfunction (ROS production and overexpression of antioxidant enzymes) [149,150]. These evidences clearly show that autophagy is induced during *T. cruzi* metacyclogenesis. On the other hand, classic modulators of autophagy that have similar effects on *T. cruzi* autophagy can modify the metacyclogenesis performance. Starvation, rapamycin, and spermidine stimulate parasitic autophagy and favor metacyclogenesis, while wortmannin inhibits this pathway and thus metacyclogenesis [143]. Interestingly bafilomycin inhibits early steps of autophagy in *T cruzi*, as evidenced by the low number of Atg8.1-positive vesicles generated under this treatment, in contrast to mammalian cells [143,151]. Spermidine is an autophagy inducer in *T. cruzi*. Overexpression of heterologous ornithine decarboxylase (ODC) allows *T. cruzi* to produce polyamines (ornithine, spermidine and spermine) and to increase basal autophagy conferring higher metacyclogenesis capacity than the auxotrophic counterpart [146,152]. In contrast, parasites overexpressing TcIP3R showed decreased metacyclogenesis [153]. Autophagy is also required during the differentiation of trypomastigotes to amastigotes since there is evidence that both starvation and rapamycin can increase this process in a similar degree that low pH, classically used to induce this differentiation [154,155].

One of the organelles involved in the autophagic pathway in *T. cruzi* is the reservosome, a late endosome-like organelle unique of trypanosomatids that concentrate endocytosed proteins and lipids. Higher content of reservosomes is found in epimastigotes while they practically disappear during differentiation into metacyclic trypomastigotes. The parasite’s main cysteine proteinase, cruzipain, is highly concentrated and active in the reservosomes and is thought to be responsible for the massive proteolysis that accompanies differentiation; since enzyme inhibitors partially block this process and overexpression of proteinases increases its rate [41]. Starvation of epimastigotes and activation of autophagy during metacyclogenesis increases the delivery of cruzipain to reservosomes. The acidity and hydrolytic activity increase in these compartments which results in the enzymatic activation and self-processing of cruzipain. Altogether these results indicate that autophagy-induced activation and self-processing of cruzipain promotes the parasite differentiation [146]. Interestingly, inhibition of cruzipain also inhibits the development and differentiation of parasites in mammalian cells, making this enzyme an interesting target of trypanocidal drugs [149].

#### Plasmodium sp.

**Figure 5.**
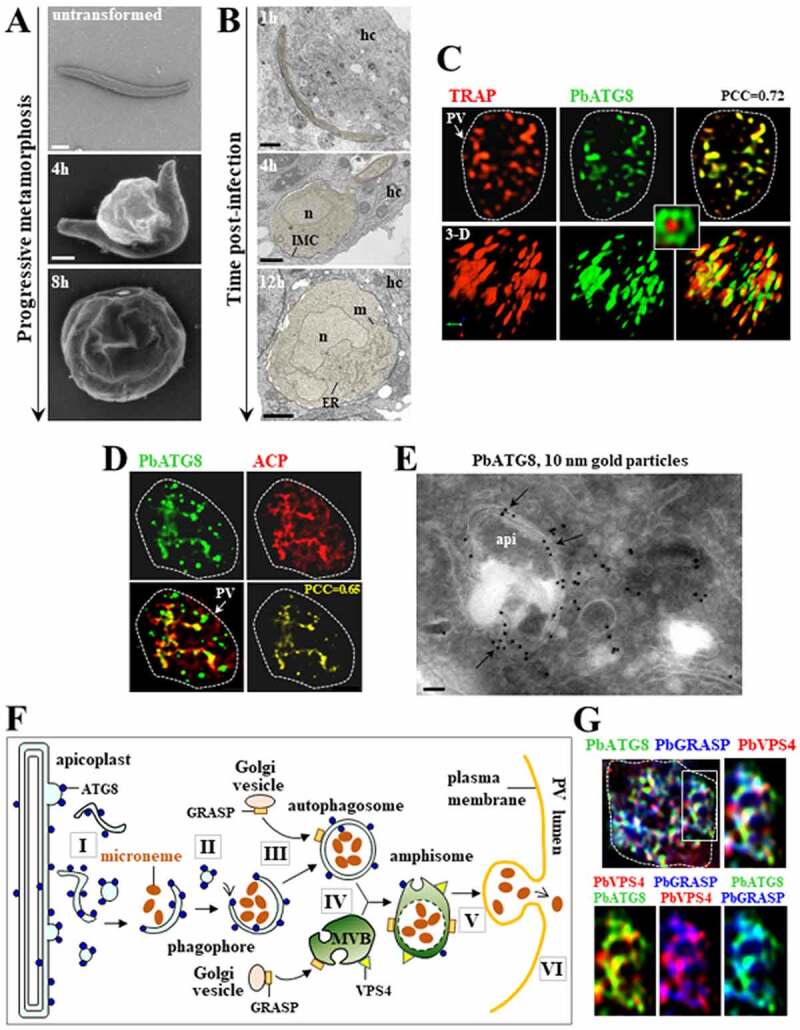
**Metamorphosis of sporozoites and microneme elimination by autophagy. (A)** Scanning EM on converting *P. berghei* maintained axenically. Bars, 1 µm. **(B)** Transmission EM of intrahepatic *P. yoelii*. Bars, 1 µm. **(C)** Double IFA using anti-TRAP and PbATG8 antibodies on intrahepatic *P. berghei* 12 h p.i., showing colocalization. **(D)** Double IFA using anti-PbATG8 and ACP antibodies on intrahepatic *P. berghei* 22 h p.i., showing colocalization. **(E)** ImmunoEM on intrahepatic *P. berghei* using anti-PbATG8 antibody, showing gold particles on the outermost membranes of the apicoplast (api, arrows). Bars, 100 nm [adapted from 160,164,169]. **(F)** Hypothetical model for microneme exophagy and amphisome detection (see text for description). **(G)** Triple IFA using anti-ATG8, anti-GRASP and anti-VPS4 antibodies on intrahepatic *P. berghei* 22 h p.i., showing significant co-association of ATG8, GRASP and VPS4 on same structures [adapted from 169].

Autophagy is a necessary event for the metamorphosis of *Plasmodium* sporozoites. Key to the parasite’s successful intracellular development in the liver is the morphologic and metabolic changes necessary for the conversion of the sporozoite (motile form) to a replication-competent (trophozoite) liver form. To invade hepatocytes, the sporozoite contains two types of secretory organelles, e.g., micronemes and rhoptries that discharge their content at the time of host cell contact. Micronemal proteins such as the thrombospondin-related anonymous protein (TRAP) contribute to hepatocyte adhesion [156,157] while the content of the rhoptries is implicated in the biogenesis of the parasitophorous vacuole (PV), a unique niche wherein the parasite multiplies in hepatocytes [158,159]. From 4 h post-invasion (p.i.) onwards, sporozoites undergo spectacular phenotypic changes in liver cells. Scanning EM to decipher the 3-D ultrastructural features of converting sporozoites reveals a bulge in the median region of the parasite that gradually expands while the two distal ends of the sporozoite retract and eventually disappear, leading to parasite sphericalization (Figure 5A). Transmission EM shows that the bulbous region corresponds to the nucleus that protrudes to the cell body (Figure 5B). This shape change is concomitant with the clearance of micronemes and rhoptries, superfluous for parasite replication. Converting parasites discharge the inner membrane complex (IMC), a specialized cortical structure composed of the plasma membrane-associated with flattened membrane cisternae and micronemes. Before release, micronemes are sequestered in double membrane-bound compartments that accumulate at the parasite periphery and are docked on the plasma membrane, suggesting exocytic events. The double membrane structures that sequester micronemes are morphologically similar to autophagosomes *Plasmodium* spp. possess a rudimentary set of autophagy-related proteins[160–163]. Transcripts of ATG3, ATG7, and ATG8 from the ATG8-complex involved in the expansion of the phagophore are detected in all *Plasmodium* stages but are upregulated in parasites upon liver infection at day 1[164]. In *P. berghei*, PbATG8 is solely membrane-associated. Double immunostaining for micronemal TRAP and PbATG8 reveals a significant overlap (Figure 5C) that increases as the conversion process progresses up to 12 h p.i., suggesting a role of the parasite autophagic machinery in microneme sequestration. PbATG8 localizes to structures that align along the apicoplast identifiable with the acyl carrier protein (ACP) marker (Figure 5D). The apicoplast is delineated by four membranes and immunogold staining reveals that PbATG8 is located on the two outermost membranes of the apicoplast (Figure 5E), which are enriched with phosphatidylinositol 3-phosphate [165], a lipid that marks mammalian autophagic structures [166,167]. PbATG8 is also found on membranes of vesicles and tubules close to the apicoplast. This suggests that the apicoplast is the source of membranes for phagophore and autophagosome formation. In *Plasmodium falciparum*, PfAtg8 was revealed to be present and expressed throughout the different sexual and asexual stages of parasite development. Strong evidence of apicoplast colocalization and branching during division was also found by immunofluorescence assays. Additionally, abolishment of apicoplast organelle through Chloramphenicol incubation resulted in disruption of PfAtg8-GFP branching during division [168]. Moreover, PfATG8 knock-down parasites were found to be lacking a functional apicoplast and failed to successfully replicate. As intrahepatic *Plasmodium* lacks degradative organelles or lysosomes [169], it is unlikely that a process of macroautophagy associated with cargo degradation in autolysosomes occurs. Alternatively, converting parasites may eliminate unwanted organelles sequestered into autophagosomal structures by a process of secretory autophagy (named exophagy), as described in other organisms during differentiation [170]. In exophagy, autophagosomes bypass fusion with lysosomes and instead fuse with the plasma membrane to release their content outside the cell. Exophagy requires the association of the Golgi protein, GRASP with autophagosomes to trigger the fusion of autophagosomes with multivesicular bodies containing VPS4 to form hybrid amphisomes that fuse with the plasma membrane [171,172]. *Plasmodium* contains genes coding for GRASP and VPS4 homologs [173,174] and GRASP and VPS4 transcripts are expressed in intrahepatic *P. berghei* 24 h p.i. [169].

Based on these data, it has constructed a working model outlining the cellular events leading to the elimination of micronemes by secretory autophagy during sporozoite conversion into liver forms (Figure 5F). In this model, the apicoplast supplies PbATG8-containing membranous structures to generate phagophores (step I); micronemes are sequestered into phagophores and autophagosomes (step II); GRASP is transported from the Golgi to autophagosomes for membrane fusion (step III); autophagosomes fuse with multivesicular bodies (MVB) to form amphisomes (step IV); amphisomes fuse with the plasma membrane for microneme release into the PV (step V) and microneme are degraded within the PV (step VI). To validate step IV of this model and illustrate an exophagic pathway intersecting with Golgi vesicles and multivesicular bodies, liver forms were immunostained for ATG8, GRASP and VPS4. VPS4-and GRASP-positive autophagic compartments were detected in converting parasites (Figure 5G). This result points to a potential cross-talk between the autophagic machinery and exocytic organelles for organelle disposal in liver forms.

To examine whether that PbATG8 plays a key role in microneme elimination by exophagy in intrahepatic *Plasmodium*, PbATG8 expression has been manipulated by creating a strain that conditionally overexpresses ATG8 (PbATG8-OE parasites [169]) when sporozoites reach the salivary glands and thus in the liver. This strain has an insertion of a 60-bp ‘GC’-rich sequence in the 3′ UTR of ATG8, resulting in the stabilization of ATG8 mRNA and a 2-fold increase of ATG8 expression during liver stage compared to the parental strain. Examination of PbATG8-OE infection in hepatocytes reveals that PbATG8-OE parasites suffer developmental defects; delayed karyokinesis and schizogony, abnormally small PV, and undersized merosomes, the membrane package produced at the end of schizogony that contains hepatic merozoites [169,175].These developmental defects of PbATG8-OE parasites in the liver lead to delays in initiating the first round of blood infection. Other abnormalities found in these parasites were expanded apicoplasts (the PbATG8 signal looks branched into a reticulate network) and delayed microneme evacuation into the PV[169]. Together, these data confirm the participation of *Plasmodium* autophagy and ATG8 in the development of the parasite infection in hepatocytes.

#### T. gondii

Like its *Plasmodium* relative, this protist parasite has a reduced ATG repertoire, but has retained some of the core elements of the autophagy systems. In this Apicomplexa, the single ATG8 homolog is involved in autophagosome formation during starvation [176,177], but like in *P. falciparum*, it is also recruited to the apicoplast along with its role to apicoplast biogenesis to ensure proper inheritance of the organelle during cell division [178]. The apicoplast is a plastid that originated from a secondary endosymbiosis event. This organelle is surrounded by four membranes, of which the outermost is decorated with TgAtg8. Although nonphotosynthetic, the apicoplast is nevertheless essential for the survival of Apicomplexa, as it is involved in several key metabolic pathways [179]. During the course of parasite replication, the apicoplast divides and must be segregated into each of the daughter cells for proper inheritance across generations. TgAtg8 seems to drive the association between the apicoplast and the centrosomes for correct positioning and segregation of the organelle [44]. The machinery for TgAtg8 membrane conjugation is involved in this original non-degradative function, as shown by defects in apicoplast maintenance and reduced cell survival observed in mutants lacking ATG3 and ATG4 [176,177]. Yet, early players in the autophagy pathway, like TgAtg9 (which is conserved in *Toxoplasma*, unlike for *Plasmodium*) is only involved in canonical autophagy, which is not essential for parasite viability in normal growth conditions in culture, but is important for surviving specific stresses [180]. Interestingly, it has been shown that this protein and thus canonical autophagy, plays an essential role in the persistence of the bradyzoite stage (the latent tissue cyst stage of the parasite)[181]. This is consistent with the fact that chemical or genetic ablation of the proteolytic capacity of bradyzoites led to autophagosome accumulation and death in bradyzoites [182]. There is much to be learned about the roles these proteins play in parasite development and can potentially shed light on the host-parasite relationships that keep these organisms thriving. *T. gondii* has not only managed to make use of its own autophagy system for organelle biogenesis, but also has brilliant ways to manipulate the host’s autophagy systems for its growth and preservation as will be explained below.

### Other (non-canonical) functions of autophagy in protists

As shown above, individual ATG proteins of protist organisms can display specialized functions other than canonical autophagy as in the case of apicoplast inheritance of Apicomplexa. That could be one reason for the diversification of some of these proteins observed in these parasites as in trypanosomatids, although the functions of homologs have not been elucidated yet. The whole autophagy process also accomplishes other non-canonical roles as will be described following.

#### Autophagic cell and nuclear death in protists

In the history of eukaryotic evolution, the concept of cell suicide was assumed to have arisen with multicellularity. However, the discovery of apoptosis in trypanosomes [183,184] and the ciliate *Tetrahymena thermophila* [185] in the 1990s indicated that a similar process of socially advantageous regulation of cell death also operates in unicellular eukaryotes. By the 2000s, apoptotic markers were described in all major groups of eukaryotes [186]. These findings highlight the utility of the altruistic form of regulated cell death in unicellular organisms for the elimination of deficient mutant or pathological individuals from the community, reminiscent of the selective elimination of unhealthy cells, such as cancer cells, for the maintenance of tissue homeostasis by apoptosis in multicellular organisms.

While most cell death involves apoptosis, studies in multicellular model systems have clearly identified instances of regulated cell death that involve autophagy. Dying cells often display a large-scale accumulation of autophagosomes and autolysosomes in the cytoplasm, which differs considerably from cells undergoing apoptosis or necrosis, and hence adopt a morphology called “autophagic cell death” or type II cell death [187]. Since promoting the use of this term in the 1980s, there have been many reports of autophagic cell death highlighting the significance of autophagy for developmental and pathophysiological cell death. Autophagic cell death has also been reported from a wide group of protists [188], implying an ancient evolutionary origin of autophagy as a suicide program that differs from apoptosis. However, genetic studies suggest that in many cases, autophagy is not directly a cause of cell death but rather accompanies apoptosis or necrosis as a failed effort to mitigate cell damage [189,190]. It is also the case that autophagy contributes to the activation of other death programs [189,190]. The term “autophagic cell death” is now used in at least three different ways based on the roles of autophagy in cell death: (i) autophagy-associated cell death, where the induction of autophagy accompanies apoptosis or other cell death pathways; (ii) autophagy-mediated cell death, where autophagy induction triggers apoptosis or other cell death modalities; and (iii) autophagy-dependent cell death (ADCD), a distinct mechanism of cell death that occurs independently of apoptosis or necrosis [191]. The recent recommendations of the Nomenclature Committee on Cell Death defines ADCD as regulated cell death that depends on the autophagic machinery without involving alternative death pathways [192]. In (i) and (ii), cell death can be mitigated by inhibition and/or genetic manipulation of autophagy mechanisms. Autophagy is however not considered as a genuine apparatus on cell death. The complex relationship between autophagy and other cell death pathways remains to be elucidated.

The strongest evidence for ADCD comes from developmentally programmed cell death in the obsolete larval midgut during *Drosophila* metamorphosis. Despite the high level of caspase activities, the removal of the midgut depends on only autophagy [193]. Autosis, a type of autophagic cell death due to activation of the Na^+^/K^+^-ATPase pump in mammalian cells [194], is also known to meet the criteria of ADCD. An additional clear-cut example of ADCD is found in the amoeba *Dictyostelium discoideum* that does not encode caspases or the major apoptotic machinery genes found in mammals[195]. Under starvation conditions, about 10^5^ vegetative amoeba cells aggregate into a multicellular organism, which then differentiates to form a fruiting body consisting of viable spores and dead stalk cells in response to cAMP production [110]. The cells forming the stalk undergo cell death with vacuolization in the cytoplasm, which is prevented by genetically blocking autophagy [196,197]. This cell death requires the morphogen differentiation-inducing factor (DIF-1) in addition to starvation[196]. Starvation alone or DIF-1 exposure to non-starved cells is not sufficient to induce cell death in the stalk. This is an intriguing example where combination of two signals converts a survival function of autophagy to a cell death promoting role. The developmental cell death in *D. discoideum* could provide an important insight into the key questions of ADCD, such as (1) how survival and lethal autophagy differ, (2) what internal/external cues promote a signal to increase the rate of flux or prolong autophagy resulting in the demise of the cell, and (3) whether ADCD involves the degradation of selective cargo or bulk degradation of cytoplasmic components, without the complexity between autophagy and apoptosis.

Autophagy, even if not ADCD, could represent a valuable drug target for parasitic protists, as it may lead to a way of autophagic cell death without side effects to the human body and damages to other animal resources. In the bloodstream-form of *T. brucei*, rapamycin, the classical inhibitor of MTOR, induces growth arrest leading to autophagic cell death [198]. Note that this effect of rapamycin on *T. brucei* is only seen at a high concentration, at least 10-fold higher than that required to induce autophagy in yeast. It remains to be determined how the autophagic events observed after treatment with high doses of rapamycin are caused and if these processes are indicative of regulated cell death by autophagy. In the mammalian-infective form of *T. brucei*, on the other hand, autophagic cell death can be induced by neuropeptides such as vasoactive intestinal peptide, urocortin, and adrenomedullin [199,200]. In this scenario, endocytosed peptides reach the lysosomes whose integrity is subsequently disrupted. The peptides are released from the late endosomes or lysosomes in the cytoplasm, where they interact with glycosomes leading to depletion of cellular ATP. The failure of energy metabolism results in cell death with involvement of autophagy. No signs of apoptosis or necrosis are observed in both the forms of *T. brucei*, implying predominant or exclusive roles of autophagy in these death processes.

In free-living protists, the freshwater green alga *Micrasterias denticulata* is an example where the role of autophagy in cell death has been investigated. In this organism, treatment with a high concentration of salt leads to cell death involving autophagosome formation, which is readily observed by transmission electron microscopy [201]. Pigment composition, photosynthesis, and respiration indicate an active metabolism, which supports a regulated form of autophagic cell death rather than necrosis [201]. Cell death in this organism can also be induced by the treatment with H_2_O_2_, but it does not display autophagosome formation [202]. The H_2_O_2_-induced cell death appears apoptotic as it shows increase in caspase-3-like activity as well as a nucleosome-sized DNA laddering[202], a key feature of nuclear degradation by apoptosis. These results show that different environmental stresses lead to different death pathways in one and the same organism, suggesting a context-specific role of autophagy in the salt-induced cell death. Note that DNA laddering also occurs in the salt-induced cell death [201]. Since neither cytochrome c release from mitochondria nor increase in caspase-3-like activity is detected[201], a caspase-independent apoptosis pathway may participate in the death process. Like the removal of salivary glands during *Drosophila* metamorphosis which requires both autophagy and apoptosis (hence not ADCD)[203], two programs may function in parallel for the salt-induced cell death, implying an additional mode of autophagic cell death.

Finally, programmed nuclear death (PND) is known to occur in the free-living ciliate *Tetrahymena thermophila* as a unique and intriguing example of autophagy. Like other ciliates, *T. thermophila* have evolved remarkable nuclear dualism that involves spatial segregation of the somatic macro- and germline micronucleus in a single cytoplasm [204]. PND is a remarkable macroautophagy process that occurs during sexual reproduction (conjugation), in which only the parental macronucleus is committed to suicide and eliminated from the cytoplasm, but other co-existing nuclei such as developing progeny macro- and micronuclei are unaffected [205]. Once there is commitment to PND, the envelope of the parental macronucleus displays an autophagosome-like property demonstrated by stainability with MDC [206] and localization of ATG5 [207] and ATG8 [208], without sequestration of the nucleus by a double membrane. Subsequently, lysosomes fuse only to the parental macronucleus to form a gigantic autolysosome for the final resorption [206]. During PND, a mitochondrial nuclease-associated protein and apoptosis-inducing factor (AIF) degrade the parental macronuclear DNA into oligonucleosomal size fragments [209,210]. These proteins contribute to the promotion of nuclear degeneration, but PND *per se* is not blocked in their knockout mutants. In contrast, loss of ATG5, ATG8, and VPS34 results in a block of the autophagy processes, rendering the retention of the parental macronucleus in the cell even after conjugation [207,208,211]. In ciliates with nuclear dualism, the parental cell-derived nucleus is taken over by the progeny macronucleus after conjugation. Therefore, PND is essential to finish the lifespan of soma and reset the developmental time of the cell to zero. The regulatory mechanism of PND appears partly different from another nuclear autophagy that is required for the removal of relic meiotic products, the haploid micronuclei not used for fertilization; loss of VPS34 does not block this autophagy [211], while ATG5 and ATG8 are prerequisite to the autophagy as well as PND [207,208]. Given the context-specific role of autophagy mediating cell death in other organisms, it will be important to investigate the regulatory mechanism of PND that acts specifically to terminate the somatic life equivalent to autophagic cell death in other protists. Each group of protists may use orphan proteins in canonical autophagic pathways and, conversely, may use conserved proteins for more specialized forms of autophagic cell death as well as PND. Understanding the commonalities and differences in autophagic cell death of protists and higher multicellular organisms will illustrate how extant autophagy has acquired its complexities and extended functions, from survival to lethality, during evolution.

### Autophagy response during drug treatment

Protist diseases that mostly affect more than 1 billion people worldwide, are mainly vector-borne diseases, have animal reservoirs and are associated with complex life-cycles, factors that make their public-health control challenging [212–216]. Moreover, due to the lack of efficient transmission control strategies and efficient vaccines against most of these diseases; in addition to limited efficacy, high toxicity and increase of drug resistance of the current chemotherapy, the development of new therapeutic interventions is imperative [214,217–221]. To further identify alternative treatments, research groups worldwide are concentrating efforts on designing novel strategies to trigger parasite death *in vitro* and *in vivo*. Besides rational drug design, other sources for drug discovery comprise natural-derived compounds and drug repositioning/repurposing [222]. As a result, several novel compounds have been found and their anti-protist activity assessed, but only occasionally further information about their mechanism of action was addressed. In this direction, mechanistic assays must be performed to identify molecular and/or cellular targets, and these findings are pivotal to guaranteeing drug safety as well as to confirm specificity or presence of off-target effects that even can be advantageous (or not) for the course of infection. Additionally, a significant number of active anti-protist compounds presented no predictable biological effect [223,224], reinforcing how crucial is the identification of the molecular targets. Interestingly, several compounds causing parasite death and proposed as potentially antiparasitic therapy seem to activate autophagy regardless of their nature. In this section we are going to summarize the autophagic response of protists to drug treatment. Besides the autophagy cell death presented previously, two other possibilities were observed: autophagy associated cell death and autophagy incidental death. In addition, a new therapeutic strategy based on targeting autophagy for vaccine and drug development will be presented.

#### Autophagy associated cell death

A couple of years ago, Jeremy Mottram’s team introduced the concept that while autophagy can be considered a regulated cell death process in mammalian cells, this type of death remains controversial for protists [188,225]. Concordantly with this notion, in most of the studies reviewed herein investigating the mechanisms involved in drug-induced parasite death, the authors observed mechanisms other than autophagy contributing to cell death and thus emphasized that this process became activated under drug stress conditions as a compensatory parasite survival mechanism in response to treatment. The autophagic phenotype, or, as introduced above, the autophagy-associated cell death is observed in almost all parasite species, independently of the drug used [226]. Evidence of autophagic activation, mainly include observation of morphological alterations via electron microscopy or the identification of autophagosomal markers [227,228].

**Figure 6.**
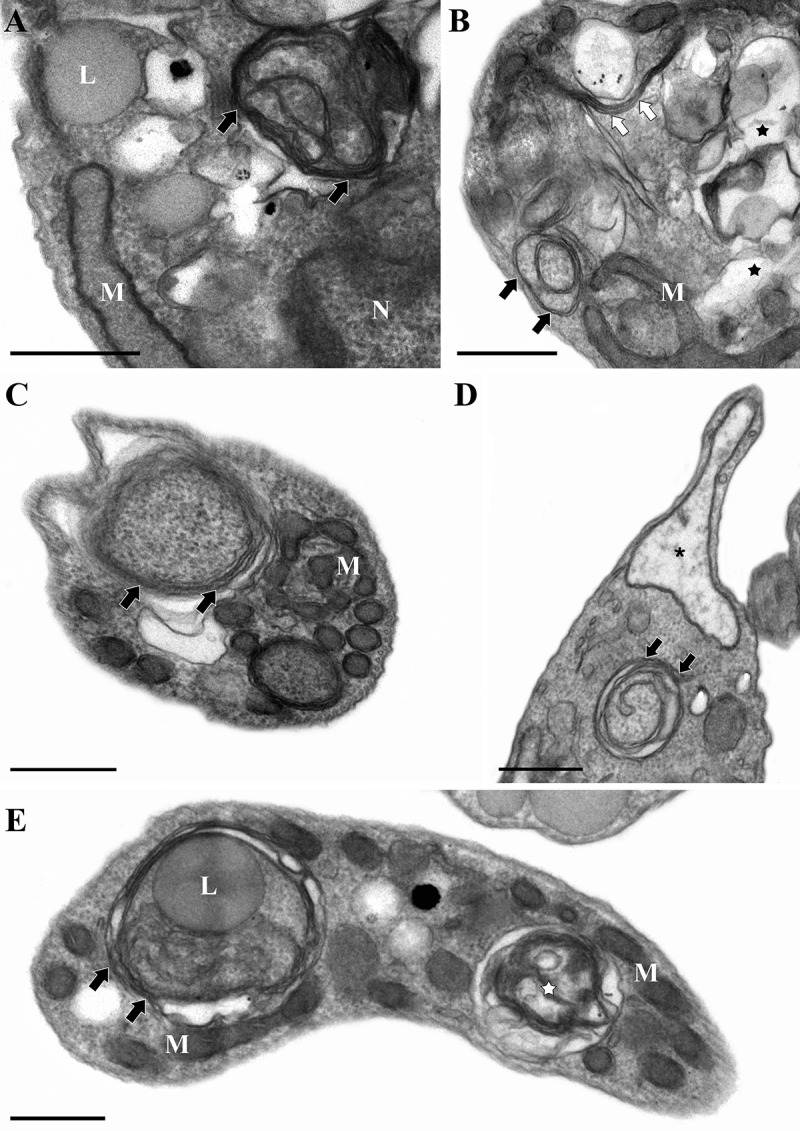
**Ultrastructural autophagic phenotype of *Trypanosoma cruzi* triggered by drugs. (A-E)** Parasites treated with different classes of drugs shared similar autophagic features such as multiple concentric membranous structures (black arrows) distributed in the cytosol, the presence of endoplasmic reticulum surrounding organelles (white arrows) as well as an intense cytosolic vacuolization (black stars) and mitochondrial swelling (black asterisk). **(E)** Increased formation of autophagosomes (white star), with distinct levels of cargo degradation is also frequently observed after the stress induction. M: mitochondrion; L: lipid; N: nucleus. Bars = 0.5 µm.

Among all approaches to the mechanistic characterization of drugs action, electron microscopy is one of the most frequently employed in almost all cellular models, including parasitic protist [221,229]. Concentric membranous structures or myelin-like figures are one of the most recurrent autophagic phenotypes detected in parasitic protist treated with all classes of compounds [228,230–239] (Figure 6A-E), followed by the high number of autophagosomes, with different degrees of cargo degradation [91,176,233–235,239–254] (Figure 6E). Another frequent morphological evidence is the appearance of remarkable ER profiles in close contact to cytosolic structures and organelles (Figure 6B). As an example, *T. gondii* treated with thiolactomycin analogues showed high amounts of expanded concentric membranes, suggestive of ER profiles [255]. In relation to trypanosomatids, elatol and amiodarone led to proeminent ER and mitochondrial swelling in *L. amazonensis* [251,256], and triazolic naphthoquinone triggered such phenotype surrounding reservosomes in *T. cruzi* epimastigotes [254].

To confirm autophagy induction, ultrastructural studies needs to be completed by tracking the autophagosome increase with specific markers. According to Klionsky and colleagues Atg8 expression is the gold standard approach for monitoring autophagy by Western blotting and fluorescence microscopy [257]. The treatment of *T. gondii* with antimalarial agents, monensin, dithiothreitol, brefeldin A or tunicamycin promoted a time-dependent increase in the number of Atg8 puncta [180,258–260]. Such increase was also observed in *T. brucei* treated with bacteriocin AS-48 and L-leucine methyl ester [239,261] as well as in cryptolepine-treated *L. donovani* as described below [253]. On the other hand, several studies still employed the non-specific probe MDC to label autophagic vacuoles. In the literature, there are many reports describing the increased number of MDC puncta in parasites treated with a great variety of compounds [143,247,249,253,262–266]^−^[46]. This is the case with *L. amazonensis* promastigotes, whose death was associated with high positivity for MDC, when treated with flavonoids [267]. The association between parasite death and autophagy was also established by enhancing MDC labeling in parasites treated with a combination of a thiosemicarbazone derivative and miltefosine [266]. Other example is antimicrobial peptides (AMPs). These compounds exhibited enhanced antileishmanial activity via a mechanism involving ionic interactions with *Leishmania*, which is modulated by parasite lipophosphoglycan (LPG). This effect results in membrane potential dissipation and the equilibration of intracellular pH with the extracellular environment. The investigators demonstrated the involvement of autophagy in parasite death, supported by observations using transmission electron microscopy (TEM) revealing cellular damage, including cytoplasmic vacuolization and the degeneration of cellular organization without the disruption of the plasma membrane, as well as the detection of intracellular vacuoles via MDC labeling [227]. Although useful as a first approach to study autophagy, MDC labeling has to be accompanied with other more specific markers to conclude the involvement of autophagy in the response of protists to parasiticidal drugs.

As an alternative approach, an experimental design including the preincubation with autophagic inhibitors, such as wortmannin and 3-methyladenine (3-MA) and subsequent treatment with the compounds has been used to assess the relation between autophagy and the parasite death induced by the drug [257,268]. In *T. gondii*, 3-MA prevented the mitochondrial effect of monensin [259], but not of different antimalarial drugs, indicating an PI3K-independent mitochondrial injury in this last case [260].

Describing the activation of the autophagic pathway in *T. cruzi* treated with naphthoimidazoles, Menna-Barreto, and colleagues [228] elegantly associated ultrastructural evidence suggestive of apoptotic-like alterations and autophagic activation with the identification of ATG gene overexpression, along with a marked increase in MDC labeling and the inhibition of death by wortmannin or 3-MA. In the epimastigotes and trypomastigotes stages of *T. cruzi*, the treatment with either one of three naphthoimidazoles N1, N2, and N3 derived from β-lapachone induces cell death. These antiparasitic compounds are known to affect the organelles of *T. cruzi*, such as mitochondria, Golgi, and reservosomes [228,269],[270]. Upon treatment, autophagic characteristics in the cytoplasm are observed; the number of concentric membrane structures stainable with MDC, increases significantly [228]. Ultrastructural studies demonstrate that the organelles, especially reservosomes, are surrounded by membrane structures like autophagosomes and lose the integrity of their structures [31,236]. The effect of the naphthoimidazoles on the cell death events is blocked by cysteine protease inhibitors, E64 and calpain I inhibitor [270], suggesting the participation of autophagic machinery in the death process as macroautophagy in mammalian cells requires calpain [271]. The preincubation with the autophagic inhibitors wortmannin and 3-methyladenine completely abolishes the effects of naphthoimidazoles and RT-PCR data indicate the participation of ATG3, ATG4, ATG7 and ATG8. These findings associated with a slight increase in phosphatidylserine exposure suggest that autophagic activation predominates in *T. cruzi* during naphthoimidazole induced cell death [228]. Eventhough, significant increases in necrosis in treated parasites support the notion that autophagy is insufficient to control drug effects in parasite death [228], indicating that the detailed pathway and proteins involved in the mechanisms of cell death require further investigation.

Cryptolepine, an indoloquinoline alkaloid that presents antimalarial and cytotoxic properties, induces *L. donovani* promastigotes to produce ROS, ultimately resulting in DNA fragmentation, a hallmark of apoptosis [253]. Alterations suggestive of apoptosis activation, including mitochondrial membrane depolarization, mitochondria swelling, increased ROS production, and phosphatidylserine’s externalization, have been described in *L. amazonensis* promastigotes treated with similar compounds, i.e., organic salts derived from quinoline[267]. The notion that autophagy functions as a survival mechanism are supported by additional decreases in the number of viable parasites in *L. donovani* cultures treated with the combination of cryptolepine and 3MA, compared to those treated exclusively with the antileishmanial drug [253]. It is possible that in response to cryptolepine, a stress response is induced by these parasites, thereby initiating an autophagic response as a survival mechanism. By contrast, treatment with quinoline derivatives alone abrogated autophagic vacuole formation, likely preventing the elimination of damaged organelles, which increased the antileishmanial effect of these compounds [267].

Altogether the findings espoused by these studies mainly support the notion that, in protists, the autophagic process is activated when submitted to stress conditions, as has been previously described in mammalian cells [272]. Thus, autophagy functions as a critical process for maintaining parasite survival under the stress produced from treatment with antiparasitic compounds [225].

#### Autophagy incidental death

In a previous work it was emphasized that until lethal signaling pathways and mechanisms associating autophagy to a death-regulated path are unveiled in protists, it is equivocal to consider cell death as distinctly different from incidental death or unregulated necrosis [225]. In line with this concept, more recently studies have consistently demonstrated that treatment of *Leishmania sp.* and *T. cruzi* with antiparasitic compounds caused abnormal activation of the autophagic pathway, subsequently resulting in incidental parasite death.

Using carvedilol, a beta-blocker broadly used to treat hypertension and other cardiovascular diseases and known to modulate autophagy in mammalian cells [273,274], Romano and colleagues [275] reported an intense accumulation of immature autophagosomes in *T. cruzi* that presented lower acidity and hydrolytic properties. Subsequently, these authors observed an intense impairment in trypomastigote survival in addition to decreased replication of *T. cruzi* epimastigotes and amastigotes *in vitro*. Furthermore, carvedilol impaired the peak of parasite burden in infected mice evidencing the effect of this treatment *in vivo*. Similar to what was observed with other compounds, ultrastructural alterations suggestive of autophagic activation were detected in *Leishmania* parasites in infected cells treated with the potent anti-cancer Hsp90 inhibitor 17-AAG, as evidenced by intense anti-leishmanial effects both *in vitro* and *in vivo* [276,277]. Moreover, transgenic parasites containing GFP-ATG8-labeled autophagosomes treated with 17-AAG exhibited autophagosomes that did not uptake cargo, such as glycosomes, nor fuse with lysosomes. Furthermore, ATG5-knockout (Deltaatg5) parasites, which cannot form autophagosomes, demonstrated lower sensitivity to 17-AAG-induced cell death than wild-type (WT) *Leishmania*, further indicating the participation of autophagy in 17-AAG-induced cell death. Interestingly, abnormal autophagic activation overloaded the ubiquitin-proteasome system (UPS). Like the UPS system inhibitor, MG132, 17-AAG also provokes a significant accumulation of ubiquitinated proteins in both WT- and Deltaatg5-treated parasites compared to controls. However, the compensative mammalian mechanism of protein aggregate formation, which is characteristic of proteasome overload, was not observed in parasites treated with the Hsp90 inhibitor. In sum, these findings suggest that overload occurs during the formation of immature autophagosomes unable to degrade their cargo, causing, consequently, incidental parasite death [278].

By previous suggestions, the present review reinforces the notion that to convincingly evidence the occurrence of regulated cell death in parasitic protists, it must be demonstrated that the death process can be delayed or abrogated by targeting essential signaling molecules or pathways. This aim could be achieved through genetic manipulation or the chemical inhibition or activation of specific pathways. We believe that such approaches should be actively employed to elucidate whether cell death can be regulated in parasitic protist and, if true, to identify the relevant mechanisms involved.

#### Targeting protist autophagy as therapeutic intervention: a new strategy of defense against Malaria

As mentioned above (see section #3), overexpression of *Plasmodium* ATG8 in liver forms result in dysregulation of autophagy leading to aberrant accumulation of micronemes and structural derangement of the apicoplast, These mutant parasites (PbATG8-OE, see above) are expected to be more sensitive to chemoprophylaxis treatment, and hence an ideal antigenic constituent of a Chemoprophylaxis Vaccination regimen (CVac).

CVac is so far the most efficient vaccination strategy using whole organisms developed against malaria and is currently being evaluated in clinical studies [168,279–282]. In contrast to radiation attenuated sporozoite (RAS) vaccination, CVac requires 20-fold fewer sporozoites to induce a sterile protective immune response and provides more robust and long-lasting protection. The immunization of mice with PbATG8-OE parasites then treated with chloroquine for 7 days after each immunization show 100% protection and remain completely blood form-negative at the memory time point of 80 days post-challenge with sporozoite wild-type in comparison with the parental strain subjected to the same protocol that reach only 60% protection [175]. These data point to PbATG8-OE parasites as a prototype of future genetically-attenuated parasites based on autophagy interference and encourage reproducing the ATG8 overexpression phenotype in *Plasmodium falciparum* to evaluate the protective efficacy of antibodies against natural *P. falciparum* infection. Additionally, designing autophagy mutants based on alterations of ATG3 or ATG7 expression levels or mutants lacking GASP or VPS4 involved in amphisome formation for microneme clearance (Figure 5) would advantageously be considered as genetically attenuated malaria parasites in case of their arrest during liver stage development.

On the other hand, ATG8 is expressed during all life stages in *Plasmodium* and is essential for parasite survival as demonstrated in ATG8 gene knockout experiments [164,283,284]. Autophagic machinery of malaria parasite cannot be substituted by other biological systems as mammalian cells or yeasts [285]. This incompatibility could then be exploited for specific interference of *Plasmodium* ATG proteins. To this point, discerned regions in the parasite ATG3 sequence could be targeted by small molecules to specifically inhibit the *Plasmodium* ATG8-ATG3 interaction and to further avoid ATG8 lipidation. PfATG3 possesses a short interacting motif of 8 amino acids (PfATG3103-110 region) involved in PfATG8 binding as demonstrated in vitro using recombinant proteins and confirmed by the production of a X-ray co-crystal structure of the PfATG3103-110 peptide and PfATG8 [286]. Importantly, PfATG3103-110 does not bind to mammalian LC3 due to the unique loop present in the *Plasmodium* ATG8 sequence (the hallmark of ATG8 in all Apicomplexa) necessary for interaction with PfATG3. Among a library of 352 small molecular fragments with an average molecular weight of 150 Da from Zenobia. Therapeutics, inhibition of full length PfAtg8 with PfAtg3 interaction was achieved by pyrogallolic acid (1,2,3-trihydroxybenzene) at low micromolar concentrations (IC50 of 150 μM) [286]. Screening of 200 drug-like and 200 probe-like molecules from the Malaria Medicine Venture Malaria Box for at least 25% inhibition of PfATG8-PfATG3 interaction at the threshold of IC50 of 5 μM identifies 6 molecules [287]. Among them, N-(4-methylphenyl)-4-pyridin-2-yl-1,3-thiazol-2-amine has been further investigated against liver stages of infection in cultured hepatocytes, and shows a 50% decrease in the proportion of cells infected with *P. falciparum* sporozoites at subtoxic concentration of 30 μM for HC-04 cells. In conclusion, targeting *Plasmodium* ATG8 lipidation is a rational strategy for drug intervention. In particular, the unique structural motifs required for *Plasmodium* ATG3-ATG8 interactions (a short interacting motif of 8 amino acids, PfATG3103-110 region [286]) have been to date amenable to chemotherapeutic interference based on in vitro studies. The promising compounds identified from libraries may then be a good starting scaffold for drug optimization, with validation of drug effect on *Plasmodium* AGT8 in animal models. More broadly, investigating many other autophagy protein interactions that may have drastic effects on normal functions of autophagy complexes could also be a starting avenue for new drug discovery against protist illnesses.

## The host autophagy during protist infections

With the exception of the ATG17 homolog that was not present in the *Homo sapiens*, mammalian cells have conserved all the repertory of autophagy proteins described in yeasts. Mammalian autophagy exhibits higher complexity than protists and displays a conjunct of functions that involve the energy maintenance in conditions of nutrient starvation, protein and organelle turnover, and its participation in cellular functions such as cell growth, cell division, and cell differentiation and cell death. However, autophagy actions in mammalian cells are not limited to these physiological roles, it is also important in pathological processes. Autophagy has a role in different diseases such as cancer, neurodegenerative disorders, and infective processes. In these pathologies autophagy can encompass opposite responses; on one side it can be beneficial for the host but also can be harmful depending on the conditions of the cells. The case of cancer exemplifies this concept: at the initial steps of carcinogenesis, autophagy contributes to eliminating malignant cells by induction of cell death but when the cancer is installed, autophagy contributes to the survival of neoplastic cells in the center of the tumor where nutrients are scarce due to low blood circulation. In the infective processes, autophagy response functions to destroy the intracellular microorganisms integrating the repertory of innate immune responses against pathogens. However, many microorganisms can subvert this host response for their own benefit, to get nutrients, or to generate membrane compartments or vacuoles with favorable environments to live in. The following sections present the current knowledge about the interaction of pathogenic protist with host cell autophagy. Given that each parasite targeted specific types of host cells, autophagy responses from phagocytes and non-professional phagocytic cells will be presented separately. Interestingly, since many of these protists possess models of infection *in vivo*, mainly displayed in mice; we added a final chapter that describes the effect of autophagy in the development of the infection in the animal model where autophagy responses are participating at different levels by regulation of cellular and even humoral immune responses.

### Infection in professional phagocytic cells

#### Autophagy during *Leishmania* infection in macrophages

Several intracellular microorganisms uniquely interact with the autophagic pathway by activating or impairing autophagy, ultimately resulting in organism survival or death [288]. This review section focuses on three main subjects approaching the knowledge regarding the role played by autophagy on *Leishmania* infection: whether *Leishmania* sp. induce autophagy during infection, how the autophagic pathway interacts with *Leishmania*-induced intracellular compartments, and how autophagy influences *Leishmania* infection outcome. During the initial steps of *Leishmania*-macrophage interaction, these parasites are recognized by a variety of receptors and internalized, leading to the formation of phagosomes containing *Leishmania* [289–295]. These acidic compartments containing lysosomal enzymes, known as parasitophorous vacuoles (PVs), are surrounded by a membrane enriched with late endosomal/ lysosomal proteins, such as RAB7, macrosialin, LAMP-1, LAMP-2, vacuolar H^+^-ATPase, and molecules of the antigen presentation machinery. Inside PVs, *Leishmania* promastigotes transform into amastigotes, which can survive and replicate, leading to infection amplification [296]. The infection outcome depends on different factors, such as parasite species and a balance between the host immune response and the parasite immune-evasion strategies[297,298]. The role played by *Leishmania*-induced autophagy on the modulation of host cell immune response has been discussed elsewhere [299–301]; therefore, this topic will not be addressed in the present review section.

**Figure 7.**
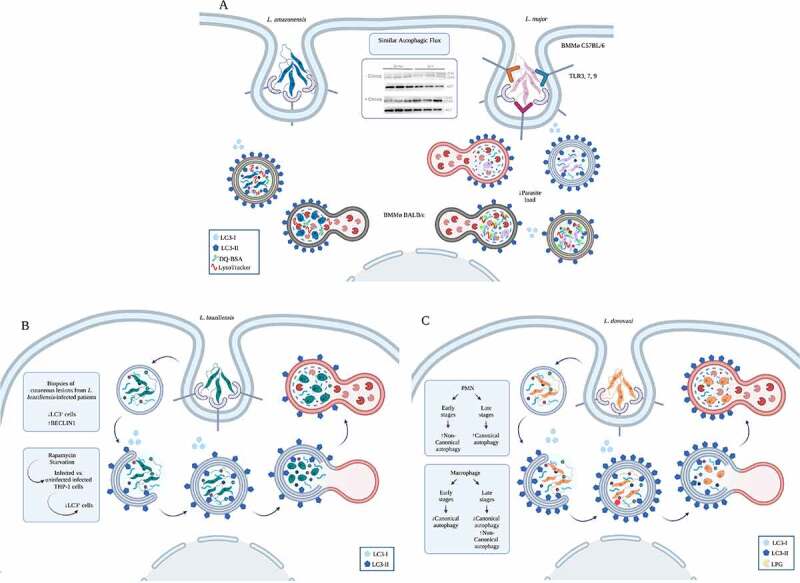
**The host autophagy during *Leishmania* infection. (A)** *Leishmania major*- and *L. amazonensis* induced autophagy. Both *L. amazonensis* and *L. major* induce autophagy in macrophages. *L. major* activation of autophagy depends on TLR3, 7, and 9, reducing parasite survival inside the host cell. *L. major*-infected macrophages present a higher percentage of parasitophorous vacuoles labeled with DQ-BSA and earlier loss of LC3-recruited molecules than *L. amazonensis*-infected macrophages. **(B)** *Leishmania braziliensis*-induced autophagy. *L. braziliensis* infection induces BECN1–Vps34 complex. This parasite also reduces LC3B expression in cutaneous lesion biopsies and a transient decrease in LC3+ cells in *L. braziliensis*-infected THP-1 compared to uninfected cells. In addition, *L. braziliensis* reduces the percentage of LC3+ cells and the number of puncta structures after rapamycin or starvation treatment in macrophages. **(C)** *Leishmania donovani*-induced autophagy. *L. donovani* induces high LC3-II to LC3-I ratios in infected THP1 cell-line, human macrophages, and PMNs. This parasite induces non-canonical autophagy in the early stages after infection and canonical autophagy later in PMNs. In macrophages, this parasite induces non-canonical autophagy at later stages of infection and inhibits classical autophagy at both early and late stages, favoring parasite survival. Created with BioRender.com.

Although labeling intracellular compartments with markers of autophagy, such as MDC and LC3 by immunofluorescence or detecting LC3 in cell extracts by western-blot indicate the occurrence of autophagy, other techniques, including TEM, the expression of other autophagy-related genes (Atg) than LC3, quantification, and detection of LC3, including accurately measuring autophagic flux, are needed to confirm autophagic activation in eukaryotic cells [191]. Regardless of the methodology employed to evaluate the autophagic activation in host cells during *Leishmania* spp. infection, data consistently supports the notion that this process occurs during most infections caused by these parasites in mammalian cells [302–309] (Figure 7).

For parasites that cause cutaneous disease, autophagic activation in response to infection seems to be unique to each *Leishmania* species. LC3 labeling by western-blot was detected in extracts of *Leishmania amazonensis*-infected macrophages from susceptible and resistant mice and in the RAW 264.7 macrophage cell line infected with this parasite[302]. Morphological alterations suggestive of autophagy activation, along with an increase in the autophagic flux, support the activation of autophagy in *Leishmania major*-infected bone marrow-derived macrophages (BMDM)[308]. Furthermore, the dependence on Toll-like receptors for the activation of autophagy in the control of *L. major* infection was proven using TLR3, -7, and -9 knockout (*Tlr3/7/9^−/−^*) mouse macrophages, which are incapable of infection control[303]. Interestingly, autophagy-induced after *L. donovani* engulfment by neutrophils is not dependent on the TLR2/4 receptor since the receptor blockade did not alter parasite lipophosphoglycan (LPG)-induced autophagy [309]. Independent of resistance and susceptibility to infection, *L. major*- and *L. amazonensis*-infected CBA macrophages similarly activate the autophagic flux, showing a similar LC3-II/Actin ratio in the extracts of infected cells [305] (Figure 7A).

The activation of autophagy in *L. braziliensis* infection was suggested by the description of the upregulation of the BECN1/Beclin1 (BECN1) – Vps34 complex, which is known to be associated with phagophore biogenesis and the maturation of autophagosomes [310], in macrophage cultures *in vitro* and biopsies of cutaneous lesions from *L. braziliensis*-infected patients. At the same time, LC3B expression was downregulated in cutaneous lesion biopsies. Immunolabeling analysis revealed a slight and transient decrease in LC3+ cells in *L. braziliensis*-infected THP-1 compared to uninfected cells. *L. braziliensis*-induced downregulation of autophagy was further supported by the impairment of the percentage of LC3+ cells and the number of puncta structures by rapamycin or starvation treatment of *L. braziliensis*-infected macrophages compared to uninfected cells. The importance of LC3B reduced expression on infection outcome was highlighted by the infection rate increase in LC3B- but not in BECN1-silenced THP-1 cells [311] (Figure7B).

One of the primary causative agents of visceral leishmaniasis, *Leishmania donovani*, induced high LC3-II to LC3-I ratios in THP1 infected cells, indicating parasite-induced activation of the autophagic pathway at later time points [304]. These findings were confirmed by a recent *in vitro* study demonstrating that *L. donovani* induces autophagy in macrophages and human polymorphonuclear neutrophils (PMNs)[307]. Consistently, greater LC3-I to LC3-II conversion was found in a human bone marrow sample from a patient with visceral leishmaniasis but not in a sample from a healthy individual [307]. Recently, Pitale et al.[309] comprehensively examined the mechanisms involved in *L. donovani*-induced autophagy, revealing this process’s complexity. While this parasite induces noncanonical autophagy at early stages after infection, it induces canonical autophagy later in polymorphonuclear cells (PMNs) infection [309]. Furthermore, although *L. donovani* induces the activation of an alternative autophagic pathway in macrophages at later stages of infection, the classical pathway was found to be inhibited at both early and late infection points in these infected cells to optimize parasite survival [304] (Figure 7C).

Interestingly, autophagic induction, demonstrated by transcriptomic analyses, induced parasite clearance of *L. major* in infected BMDM in the late phase of infection [308]. One possible explanation for autophagy distinctly influencing infection outcome *in vitro* relies on the recent description of differences in the parasitophorous vacuoles’ (PVs) autophagic features induced by *Leishmania* sp. Indeed, compared with mouse macrophages from CBA mice infected with *L. amazonensis, L. major*-PVs presented a double percentage of vacuoles labeled with the degradative compartment marker DQ-BSA. In addition, LC3-II is lost earlier in *L. amazonensis* than in *L. major* PVs, although both compartments were labeled similarly by LysoTracker, an acidic compartment marker [305]. Notably, autophagy inhibition by a specific VPS34 inhibitor, VPS34-IN1, or activation by rapamycin did not alter the degree of LC3 recruitment to *L. amazonensis*- or *L. major*-induced PVs. Similarly, rapamycin-induced autophagy did not modify LC3 labeling in *L. donovani*-infected THP-1 cells compared to untreated cells[304]. In contrast, LC3 positivity and the number of puncta structures were impaired in *L. braziliensis*-infected THP-1 cells compared to the control, untreated macrophages [311]. These findings also reinforce the notion that during infection, autophagy can be activated via a pathway other than PI3K-Akt-mTOR.

Contradictory results regarding the role played by autophagy in the *Leishmania* infection outcome probably arise from the type of methodology used to investigate this effect. Thomas et al.[304] showed that the abrogation of autophagic activation by a knockdown of Atg5 and Atg9 in THP-1 cells decreases intracellular *L. donovani* survival. Furthermore, during *L. amazonensis* infection, starvation causes differing outcomes depending on the strain of infected mouse macrophages [312]. The intracellular viability of *L. amazonensis* is increased in macrophages from susceptible BALB/c mice but not in those from resistant mice [312]. This effect was not observed for *L. major* in BALB/c mouse macrophages. In contrast, using a more specific methodology, autophagy abrogation secondary to Atg5 knockdown in BALB/c[308] and C57BL/6 [303] macrophages increased *L. major* intracellular viability. Interestingly, autophagic activation before infection profoundly affects macrophage phagocytic capacity [313,314], suggesting that inhibition of autophagy should be made after infection by using a genetic approach or plasmids with inducible promoters. Employing another mouse model, in CBA/j mouse macrophages that were proven resistant to *L. major* yet susceptible to *L. amazonensis*, physiological and pharmacological inhibition of autophagy did not alter either *L. amazonensis* or *L. major* parasitic load. On the other hand, autophagic induction significantly increased *L. major* intracellular viability within PVs that notably exhibited a more degradative feature than those induced by *L. amazonensis* [305].

In sum, the discrepancy involving *Leishmania*-induced autophagy in host cells should be counteracted by performing studies using complementary comparative methods for expanding the identification of common targets from the autophagic pathway for an appropriate design of new therapeutic strategies in treating leishmaniasis.

#### LC3-associated phagocytosis during *Leishmania* infection

As key players of the innate immune system, professional phagocytes recognize, engulf, and destroy pathogens through a process known as phagocytosis. Here, pathogens are internalized in a vacuole, the phagosome, which undergoes a complex maturation program characterized by a series of highly regulated sequential interactions with diverse cellular compartments [315]. These interactions, which are essential for the generation of a microbicidal phagolysosome, entail the accrual of numerous proteins, including the vacuolar proton (H^+^)-pumping ATPase (V-ATPase) and of an array of degradative hydrolases to these organelles [316,317]. Most microbes are destroyed in such a degradative environment, leading to the generation of peptides through the degradation of microbial antigens. These peptides are loaded on MHC molecules and transported to the cell surface to initiate an adaptive immune response [316,318–320], establishing a link between innate and adaptive immunity [316,321]. Phagolysosome biogenesis thus represents an important means of controlling infections.

A non-canonical autophagic pathway known as LC3-associated phagocytosis (LAP) provides a link between phagocytosis and autophagy [322–325]. The salient feature of LAP is the recruitment of the autophagy-related protein LC3 to phagosomes, which takes place early on during the internalization process [322]. Hence, time-lapse analysis of LC3 association to phagosomes indicated this event occurs rapidly, within 15 min after internalization and is transient, as LC3 starts dissociating from phagosomes after 60 min [322]. Consistent with its role in host defense against pathogens, including bacteria, fungi, and parasites [324], LAP requires the triggering of various pattern recognition receptors including toll-like receptors, Dectin-1, Dectin-2, as well as receptors for phosphatidylserine for the recognition of apoptotic cells [322,326–328]. Although the significance of LC3 recruitment on phagosomal functions remains to be fully elucidated, LAP is characterized by an enhancement of phagosome maturation and microbial killing [322], as well as of MHC class II-mediated antigen presentation [328].

One of the key features of LAP is the essential role of NADPH oxidase (NOX2)-mediated production of reactive oxygen species (ROS) in the recruitment of LC3 to phagosomes [329]. ROS generated by NOX2 play a central role in the biology of phagocytes, including microbial killing and antigen cross-presentation [328,330,331]. Assembly and activation of the NOX2 complex to phagosomes during LAP is a highly regulated process. Upon stimulation of pattern recognition of receptors by pathogens or apoptotic cells, the cytosolic components of NOX2 p40*^ph^^ox^*, p47*^ph^^ox^*, and p67*^ph^^ox^* form a heterotrimer which is then translocated to the phagosome membrane to assemble with the membrane-associated flavocytochrome *b*_558_ components gp91*^ph^^ox^* and p22*^ph^^ox^*[330]. Although LAP requires a number of components associated with canonical autophagy [324], it remains distinct for a number of characteristics. In particular, LAP necessitates the activity of Rubicon, which controls the phagosomal association of a structure known as the LAPosome [332]. In addition, Rubicon is essential for the phagosomal assembly and activity of NOX2, production of ROS, and recruitment of LC3 to phagosomes [332]. Phagosomal assembly of NOX2 is also regulated by a number of modulators of membrane fusion, including Synaptotagmin XI, VAMP8, and SNAP23 [333–335]. Hence, membrane trafficking mechanisms associated with these SNAREs play an important role during LAP [306,335].

Given the potentially lethal consequences of exposure to toxic ROS, it is not surprising that a number of intracellular pathogens evolved virulence strategies to interfere with phagosomal assembly of NOX2 [336–340]. To establish infection within a hospitable intracellular compartment, both developmental stages of the *Leishmania* parasite prevent assembly of NOX2 at the phagosome through various strategies. Hence, amastigotes of *L. pifanoi* induce heme degradation through the activation of heme oxidase-1, preventing maturation of the immature form of gp91*^ph^^ox^* and ROS production at the phagosome [341]. Amastigotes of *L. donovani* evade serine phosphorylation of p47*^ph^^ox^*, impairing the recruitment of both p47*^ph^^ox^* and p67*^ph^^ox^* to phagosomes and ROS generation [342]. Promastigotes use at least two distinct strategies to prevent phagosomal assembly of a functional NOX2. One involves inhibiting the recruitment of the cytosolic p47*^ph^^ox^*/p67/p40*^ph^^ox^* heterotrimer to the phagosome through the action of the cell surface virulence gycolipid lipophosphoglycan (LPG) [343]. The second strategy consists in the cleavage of the SNARE VAMP8 by the virulence-associated metalloprotease GP63. By cleaving VAMP8, *Leishmania* prevents NOX2 assembly to the phagosome and the recruitment of LC3 and inhibits LAP [306]. Importantly, inhibition of LAP may require live parasites, as apoptotic *L. major* promastigotes are readily internalized by macrophages in LC3-positive compartments via LAP [344]. Of note, in contrast to the phagocytosis of other types of particles, *Leishmania* does not trigger the recruitment of BECN1 to phagosomes [306]. Interestingly, induction of host macrophage autophagy by *L. braziliensis* is BECN1-independent [311]. Thus, both amastigote and promastigote forms of *Leishmania* use a panoply of strategies to prevent phagosomal NOX2 assembly/activity which, in addition to protecting the parasite from toxic ROS, leading to the impairment of the microbicidal LAP process in the host phagocytes.

### Infection in non-professional phagocytic cells

In contrast to *Leishmania sp*., which subverts phagocytosis to infect host cells and thus develop only on professional phagocytic cells, *T. cruzi* and *T. gondii* exploit many mechanisms to invade cells and therefore can invade both professional and non-professional phagocytes. The effect of host autophagy on the outcome of infection is not always the same for both types of cells and contradictory data can be found in the literature about this matter. Interestingly, although both *T. cruzi* and *T. gondii* establish their replicative niches in non-professional phagocytic cells, as myocytes, brain and eyes, they can also infect macrophages, neutrophils and dendritic cells at initial times of infection or to gain access to immunologically privileged tissues such as brain in neurotoxoplasmosis. In contrast, *Plasmodium* species target hepatocytes and red blood cells establishing its infection only in non-professional phagocytes.

#### Different phenotypes are displayed by host autophagy during *T.* *cruzi* infection

**Figure 8.**
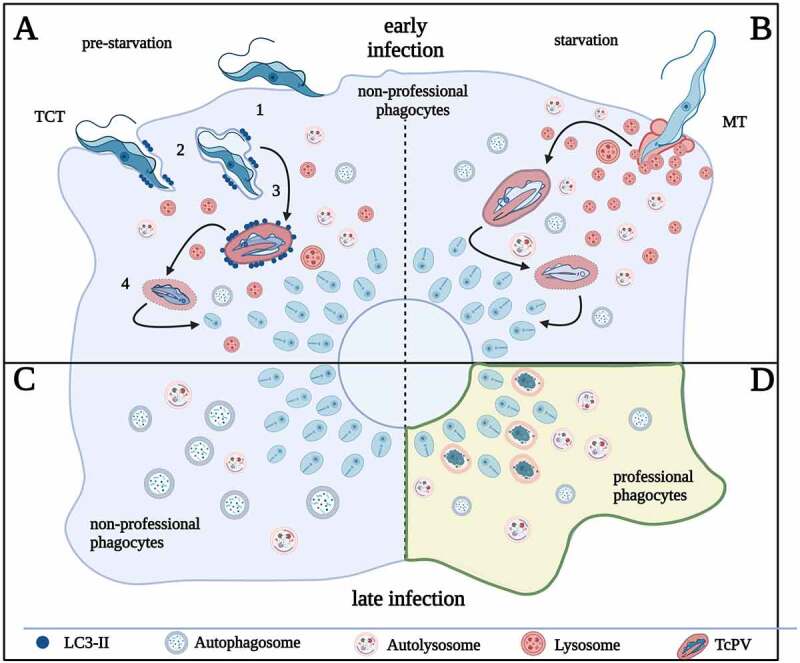
**The host autophagy during *T. cruzi* infection. (A)** invasion of tissue cell trypomastigotes (TCT) is characterized by 4 steps, adhesion of parasites to cell surface (step 1), internalización/parasitophorous vacuole formation (step 2), vacuole maturation (step 3) and exit of parasites to cytosol by vacuole membrane disruption (step 4). TCT vacuole is decorated with LC3 and infection increase in pre-starved cells by favoring the vacuole maturation. **(B)** MT invasion is characterized by plasma membrane wounding and lysosomal exocytosis. Starvation of cells favors infection of MT by increasing lysosomal formation and scattering. **(C)** *T. cruzi* infection increases autophagosome number. Amastigotes developed at later times of infection inhibit autophagy flux in non-professional phagocytic cells. **(D)** In professional phagocytic cells (or in cardiac cells treated with autophagy inducers), amastigotes are targeted by xenophagy and destroyed. Created with BioRender.com.

Depending on the stage in the biological cycle, two infective forms of *T. cruzi* can interact with mammalian host cells, the tissue-derived trypomastigotes (TCT), equivalent to bloodstream trypomastigotes, produced by differentiation of amastigotes in the host cells, and the metacyclic trypomastigotes (MT) present in the insect vector. For TCT, the initial events during the *T. cruzi* intracellular cycle can be divided in 4 main steps [155,345]: (1) Adhesion of trypomastigotes to the host cell surface; (2) invasion/internalization of trypomastigotes and formation of the *T. cruzi* parasitophorous vacuole; (3) maturation of PV by fusion with lysosomes; and (4) exit of parasites from PV to cytosol and differentiation into amastigotes (Figure 8A). After that, amastigotes replicate several times until they transform back into trypomastigotes which exit the cell to start a new infective cycle. As *T. cruzi* can infect all nucleated cells, the host response and the parasite life-cycle in those cells can be peculiar. Features such as different (i) parasitic forms and their source (metacyclic, bloodstream, or tissue culture trypomastigotes (TCT)); (ii) strains; and (iii) host cell type directly influence the parasite-host interaction. In the first description on host autophagy in *T. cruzi* infection, Romano and colleagues showed LC3 recruitment to the parasitophorous vacuoles (PVs) of TCT from CL Brener strain in a non-phagocytic cell line (CHO: Chinese hamster ovary cell line) [154] (Figure 8A). This recruitment on the PV was also observed in other *T. cruzi* strains and host cell types such as cardiac-derived cell lines and RAW macrophages [346]. The induction of host autophagy (by starvation or rapamycin treatment) before the infection led to an increase in the number of infected cells at early times after infection but not variation in the amastigote replication occurred when autophagy was induced after the infection, at later times [154]. Inhibitors of canonical autophagy such as wortmannin, 3-methyladenine or vinblastine, and the inhibitor of the spermidine biosynthesis, DFMO (difluoromethylornithine) reverted the effect of starvation and decrease the LC3 recruitment [154,347]. Acquisition of the endosomal/lysosomal markers Lamp-1 and LBPA to PVs increased at starvation conditions suggesting that autophagy induction promoted the infection by favoring the previously described lysosomal dependent entry of *T. cruzi* in the host cells [348] or by promoting the fusion of lysosomes with the early vacuole, a key step for retention of *T. cruzi* trypomastigotes inside host cells [349]. A more recent work showed that *T. cruzi* (Tulahuen strain) increases the number of LC3 dots after infection in a human fibrosarcoma cell line [350]. Increased number of LC3 vesicles was also observed in CHO cells infected with the Y strain of *T. cruzi* together with the formation of LC3-positive tubules, an interesting feature not found when these cells were treated with rapamycin and whose relevance will need to be explored in the future [155]. Working with metacyclic trypomastigotes of CL strain, Dr. Yoshida laboratory found that starvation of HeLa cells at the same time of *T. cruzi* infection increases the lysosome formation and scattering required for the invasion of MT to host cells, while TCT enter preferentially by a lysosomal-independent process that is benefited when host cell starvation is made prior infection [351] (Figure 8A and B). Together, these evidences showed that starvation increases *T. cruzi* infection in a manner that depends to the mechanism exploited by *T. cruzi* to infect cells, by the lysosomal-dependent or independent (endocytic-like) route. In the same way interaction of *T. cruzi* with host cell autophagosomes and maturation of the *T. cruzi* vacuole by the acquisition of lysosomal markers are differently modulated according to mammalian cell and parasite features. Time of infection is also important. At later times when amastigotes are present in the cytoplasm of host cells, LC3 recruitment to these parasites was very low at basal autophagy conditions [154,350]. Although *T. cruzi* infection increases the number of LC3-positive vesicles, Onisuka and colleagues showed that these autophagosomes did not acquire syntaxin-17, a SNARE protein associated with autophagosome-lysosome fusion, and lysotracker, suggesting that *T. cruzi* can inhibit the autophagy flux to evade autophagic capture and degradation, corroborated by non-degraded p62, an autophagy cargo receptor [350] (Figure 8C).

Quite different is the action of autophagy on the *T. cruzi* infection in phagocytic cells. In this case it was observed that autophagy response results in the *T. cruzi* elimination by xenophagy of amastigotes at later times of infection [352]. This selective process increases in the presence of autophagy inducers such as ursolic acid [352] and has the opposite effect in the presence of autophagy inhibitors or in the BECN1 heterozygous KO peritoneal cells [353]. Furthermore, other studies corroborate that autophagy displays not only a direct action in the parasite but this process interlinks other host processes to subvert infection in primary culture cell models. In activated C57Bl/6 peritoneal macrophages, infection induced LC3 and p62 expression, and rapamycin decreased the number of infected cells and parasite proliferation in a NLRP3-dependent pathway [354]. Similar data were obtained in peritoneal macrophages and cardiac cells infected with bloodstream trypomastigotes from the *T. cruzi* Y strain, whereas starvation induced after infection decreased the infection only in cardiac cells, indicating different roles of autophagy regarding cell type [355] (Figure 8D). Also in this study, infected *Atg5*^−/−^ MEF showed an increased percentage of infection [355]. As it was mentioned above, different host cell and parasite models, as well as distinct experimental designs, could lead to the variety of autophagic phenotypes of *T. cruzi* infected cell previously reported, emphasizing the huge repertory of molecules involved in the parasite-host cell relationship. This hypothesis is strengthened by the substantial differences in surface protein repertoire between tissue culture, bloodstream, and metacyclic trypomastigotes from the Y strain [351,356]. Those molecules, that modulate several processes during *T. cruzi* entry such as parasite adhesion, host cytoskeleton remodeling, and lysosome location, could explain the different results obtained in each model. Systematic comparative studies combined to the best technology available, which is another important source of data variation, will need to be conducted in the future to explain the differences found.

#### Host autophagy during *T.* *gondii* infection

*Toxoplasma gondii* uses an active invasion process to infect many kinds of nucleated mammalian cells, including professional and non-professional phagocytic cells. The process involves the formation of a receptor-ligand mediated tight apposition of the parasite and host plasma membranes known as the moving junction [357]. As the moving junction translocates from the apical to the posterior end of the parasite, it excludes host transmembrane proteins [358], thereby forming a specialized intracellular compartment known as the PV [359]. Excluding host transmembrane proteins renders the PV non-fusogenic [360], thus allowing the parasite to avoid degradation by lysosome fusion, at least initially. Thereafter the parasite secretes lipids and proteins to modify the PV for its replication.

Host cell defense against *T. gondii* infection involves the autophagy machinery. As early as 2 min post-infection, host LC3 or homologs (GABARAPs) are detected at the surface of *T. gondii* PV, indicating the detection of the intruder by host autophagy. Lipidated LC3 is directly incorporated *in situ* in the PV membrane (PVM) and not by the fusion with an LC3-positive autophagosome, indicating the occurrence of a LAP-like process different from canonical autophagy. LC3 lipidation in *Toxoplasma* infected cells is mediated by the complex ATG5-ATG12-ATG16L1 and the enzymes ATG7 and ATG3. Although the nature of the factor(s) to recruit ATG12-ATG5-ATG16L1 complex to the PV is unknown, it is possible that abnormal membranous structures or a pathogen-associated molecular pattern (PAMP) participate in this recognition. Downstream of LC3, two different mechanisms that mediate parasite killing and control toxoplasmosis have been described. The autophagy-dependent killing of parasites is activated by CD40 and its main ligand CD154[361]. CD40 activation induces recruitment and fusion of host lysosomes to LC3-positive PV followed by parasite destruction [362]. The second process is triggered by interferon-gamma (IFN-γ). IFN-γ is a pleiotropic cytokine that plays a key role in activation of cellular immunity and antitumor immune-response. This protein has a main role against *T. gondii* in hematopoietic and non-hematopoietic cells [363]. IFN-γ induces the localization of IFNγ-regulated GTPases (IRGs) and guanylate-binding proteins (GBPs) to *T. gondii* vacuoles [364,365]. These proteins vesiculate the PVM of *T. gondii*, thereby exposing the denuded parasites to autophagosomes [364,366]. Loading of IRGs on the PVM depends on the prior recruitment of the autophagy proteins ATG3, ATG7 and ATG5-ATG12-ATG16L complex, but not LC3 [366,367]. Also, activation of Arf1 by GABARAPL2 (GATE16) enables GBP to remain in a non-aggregated form, thus promoting GBP loading onto PVM [365]. These mechanisms were observed mainly in mouse-derived cells. In human epithelial cells, ubiquitin, the autophagy receptors NDP52 (nuclear dot protein 52) and SQSTM1/p62, and LC3 are deposited on *T. gondii* PV by the action of an interferon-stimulated gene 15 (ISG15)-related pathway. This produces the entrapment of the PV by a multilayer structure that restricts parasite growth [368]. In human endothelial cells infected with type-II strains, loading of ubiquitin, SQSTM1/p62 and NDP52 on the PVM is followed by acquisition of Rab7 and fusion of PV with lysosomes leading to parasite destruction [369]. Because this process does not involve ULK1, ULK2, or Atg14L, and classic modulators of autophagy (rapamycin, wortmannin, chloroquine, bafilomycin A1) have no effect, most authors agree that IFNγ functions to limit *T. gondii* infection in this context via non-canonical autophagy [369–371]. Although both CD40 and IFNγ toxoplasmacidal mechanisms share some molecular components, there are specific differences between them. CD40 activation induced *T. gondii* killing by autophagy via ULK1, BECN1, PI3KC3, ATG5, ATG7, and lysosomal enzymes [367,372] whereas ULK1 and BECN1 do not participate in IFNγ activated parasite death. In addition, interferon-dependent autophagy is selective for type II parasites, while CD40-induced autophagy kills type I and II parasites [370,371,373,374]. Despite this arsenal of autophagy effectors and IRG at the PV to control infection, infected host cells remain incapable of clearing all parasites. Only a small portion of *Toxoplasma* PVs (less than 10%) are targeted by LC3. This means that *T. gondii* has evolved successful strategies to subvert the destructive actions of host autophagy. One strategy *Toxoplasma* uses involves stimulating the host epidermal growth factor receptor (EGFR)/PI3K/Akt signaling axis. Extracellular *T. gondii* release proteins (MICs) from apical secretory organelles (micronemes) to support gliding motility and facilitate cell invasion. Several MICs contain multiple domains with homology to EGF [375] that act as ligands of EGFR. MIC/EGFR engagement triggers EGFR autophosphorylation, resulting via several steps in the phosphorylation and activation of the kinase Akt, which in turn activates MTOR to suppress host autophagy [376]. Besides this inhibition of the defense mechanisms of host cells, *T. gondii* takes advantage of host cell degradation pathways to acquire a sustainable source of nutrients for growth [377]. Microscopic observations showed the particular location of PV containing replicating parasites between the endocytic and secretory pathways. Indeed host endocytic organelles and Rab-positive vesicles are observed in the PV lumen [378]. Non-selective autophagy also benefits the parasite, as another source of nutrients. On one hand, it has been shown that proteins (ROPs) secreted from apical rhoptry organelles can modulate host canonical autophagy. ROP17 interacts with Bcl-2, disrupting Bcl2-BECN1 interaction, and increasing LC3 and p62 expression [379]. On the other hand, ROP16 contributed to Focal Adhesion Kinase/Signal Transducer and Activator of Transcription 3 (FAK/STAT3) activation, impairing autophagy clearance [380]. The induction of host autophagy also impacts *T. gondii* infection. In human embryonic fibroblasts, rapamycin increased infection [145], nevertheless, in Vero cells and other human fibroblasts, starved cells induced parasite cell death by mitochondrial fragmentation [240]. In primary skeletal muscle cells, autophagy inducers reduced infection [242]. Using human umbilical cord mesenchymal stem cells to mimic congenital toxoplasmosis *in vitro, T. gondii* downregulated MCL-1, an anti-apoptotic protein, disrupting BECN1 interaction, promoting autophagy and apoptosis by caspase-3-cleavage [381]. Additionally, the parasite uses fatty acids from host lipophagy for its growth, competing with the host mitochondrial network [382].

**Figure 9.**
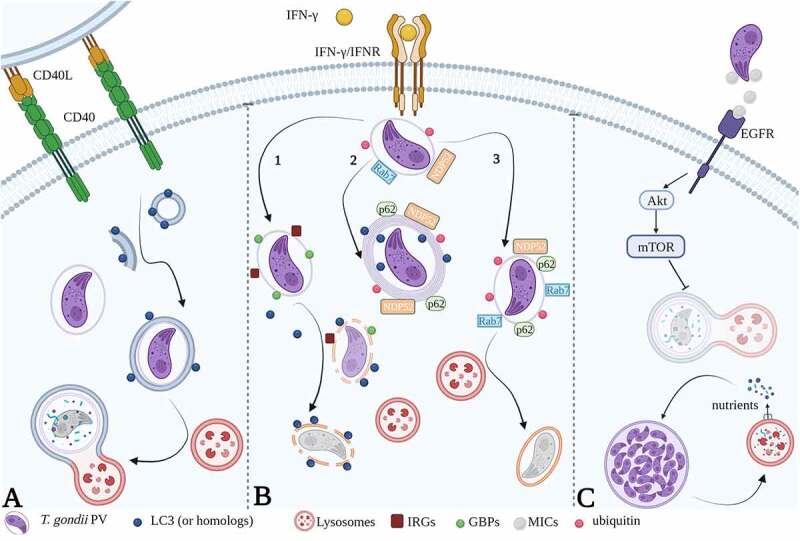
The h**ost autophagic responses during *T. gondii* infection. (A)** activation of host cell autophagy by CD40 stimulus induces the fusion of *T. gondii* PV with lysosomes followed by parasite destruction. **(B)** In murine cells, IFN-ɣ induces the recruitment of IRGs and GBPs on the PVM which expose the denuded parasites to destruction by autophagosomes (pathway 1). In human epithelial cells, ISG15 induces the ubiquitination of PVM followed by SQSTM1/p62 and NDP52 mediated LC3 recruitment. Concentric LC3-positive membranes around PV limit parasite replication (pathway 2). In human endothelial cells, ubiquitination and loading of SQSTM1/p62 and NDP52 are followed by Rab7 recruitment and lysosomal fusion leading to parasite destruction (pathway 3). **(C)** *T. gondii* resists host cell toxoplasmicidal action by inhibiting selective autophagy via MICs-dependent EGFR activation. Induction of canonical autophagy and lipophagy provide nutrients for *T. gondii* growth. Created with BioRender.com.

In conclusion, host cells resort to different innate immunity and cell-autonomous mechanisms to contain the parasitic infection. Some of these mechanisms encompass the activation of the canonical autophagic pathway (Figure 9A), and others that use the ubiquitination machinery (Figure 9B). They turn the PV into a fusogenic or damaged vacuole finally destroyed by the lysosomal degradation pathway. For its part, *T. gondii* has evolved to limit the activation of autophagy and subsequent lysosomal degradation (Figure 9C). The effect of this pathway is dependent on the host cell type and explains why under certain conditions *T. gondii* induces autophagy and uses it for its own benefit.

#### Host cell autophagy-related pathways during *Plasmodium* liver stage infection

**Figure 10.**
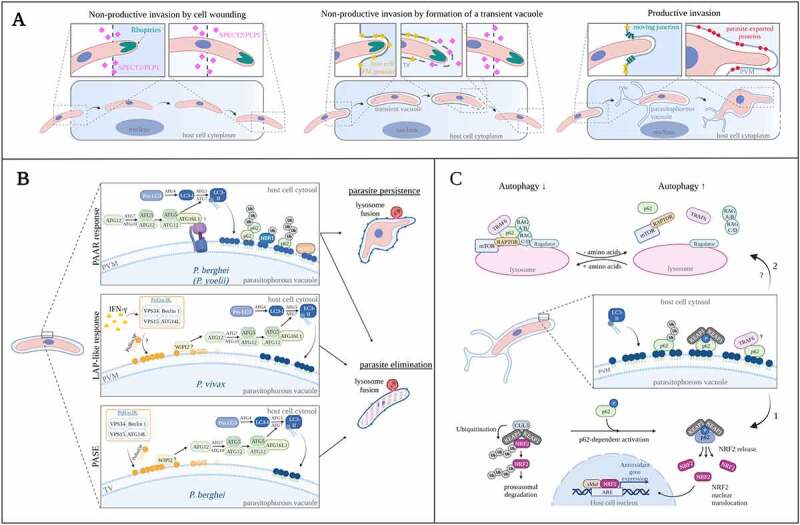
**Cell invasion by *Plasmodium* and autophagy associated host responses. (A)** Different types of invasion: non-productive invasion by cell wounding, non-productive invasion by formation of a transient vacuole, and productive invasion. **(B)** Mechanisms of host response against *Plasmodium*: the *Plasmodium*-Associated Autophagy-Related (PAAR) response targeting *P. berghei* parasites, the LAP-like response against *P. vivax* parasites, and the PI3P-Associated Sporozoite Elimination (PASE) against *P. berghei* parasites trapped in a transient vacuole. The molecular components involved in each response were depicted (see text for description). **(C)** Signaling functions of SQSTM1/p62. Middle panel: SQSTM1/p62 is recruited by LC3 and associates with the parasitophorous vacuole membrane of *Plasmodium* liver stage parasites. Upper panel: p62 interaction with TRAF6 is essential for MTOR activation and suppression of canonical autophagy (pathway 2). Bottom panel: binding of KEAP1 to phosphorylated p62 increases NRF2 activation. NRF2 is stabilized and translocates into the nucleus to induce the expression of cytoprotective genes that benefit *Plasmodium* (see text for description). INF-γ: interferon-gamma, PLP1: perforin-like protein 1 (also called SPECT2), PV: parasitophorous vacuole, TV: transient vacuole, PVM: parasitophorous vacuole membrane, TVN: tubovesicular network. Created with BioRender.com.

Sporozoites, the infective form of *Plasmodium* for the vertebrate host, exhibit gliding motility[383], a substrate-dependent locomotion depending on their actin myosin motor located between the inner membrane complex and the parasite plasma membrane [384]. Eventually, they cross the endothelial barrier by either transmigration of Kupffer cells, endothelial cells or paracellularly between two cells [385]. Cell traversal (CT) is not only important for reaching the liver, but also inside the liver parenchyma. Sporozoites first transmigrate several hepatocytes until they finally invade one productively [386]. CT can occur either by disrupting the plasma membrane of the traversed hepatocytes and moving freely in their cytoplasm (Figure 10A, non-productive invasion by cell wounding) or by forming transient vacuoles (TVs). TVs are formed by invagination of the hepatocyte’s plasma membrane when sporozoites transmigrate the cell without forming a moving junction and are subsequently disrupted [387](Figure 10A, non-productive invasion by formation of a transient vacuole). In contrast, productive invasion of *Plasmodium* sporozoites is characterized by an invagination of the host cell plasma membrane forming a specialized and stable compartment, the parasitophorous vacuole (PV), in which the parasite resides (reviewed in Loubens M, et al 2021 [388]). Proper PV formation requires the establishment of a moving junction complex between the invading parasite and the host cell at the site of entry [357]. The moving junction acts as a molecular sieve, excluding host cell proteins from the emerging PV membrane (PVM)[389,390]. Therefore, a fully functional PVM, which initially does not contain typical host cell plasma membrane proteins, differs from a TV membrane, which contains all host plasma membrane proteins present at the time of parasite entry (Figure 10A, productive invasion). Export of proteins and maintenance of PVM integrity are crucial for successful liver stage development. Interestingly, parasites that exhibit problems with PVM formation or maintenance are commonly subject to elimination, underlining the importance of the PVM in maintaining a reproductive niche for the parasite [391–394]. A functional PVM not only promotes the uptake of nutrients, but also the release of waste products into the host cell and most likely it acts also as a signaling hub between host cytosol and parasite. The PVM contains pores which allow passive diffusion of small molecules up to about 855 Da [395]. PVM modifications include parasite protein export to the PVM and a change in the membrane’s architecture by formation of a tubovesicular network (TVN)[395–398].

Thus, host cells are confronted with parasites residing in different types of vacuoles and have to react adequately to control infection.

During their development in hepatocytes, *Plasmodium* parasites face major challenges. On one hand, they require considerable amounts of nutrients and lipids for their extensive growth. On the other hand, parasites must escape host cell defense mechanisms. There is increasing evidence that autophagy-related responses play a role in both aspects. This means that host cell autophagy during the *Plasmodium* liver stage can have beneficial as well as deleterious effects on the parasite. It is therefore important to distinguish between starvation-induced canonical autophagy, which can supply nutrients and autophagy-related pathways as an intracellular immune response against the parasite. The liver stage *Plasmodium* parasite is one of the fastest growing eukaryotic organisms [399]. Therefore, it needs a constant supply of nutrients which must be provided by the host cell (reviewed in Nyboer B, et al 2018 [400]). Starvation-induced canonical autophagy represents an important source of nutrients for the growing parasite. Inhibition of canonical autophagy by genetic manipulation of *FIP200* and *ATG5* lead to a reduction in parasite growth [401–403]. This growth defect could be partly compensated by amino acid supplementation of infected *ATG5*^−/−^ cells [401]. These data, also supported by *in vivo* experiments (see below), depict the positive role of host canonical autophagy on parasite growth. Defense action of autophagy-related pathways against the parasite will be explained in the following paragraphs.

All *Plasmodium* species analyzed until now are recognized via non-canonical autophagy mechanisms that are independent of the ULK complex and the nutritional status of the cell. These mechanisms are: the *Plasmodium*-Associated Autophagy-Related (PAAR) response; the LAP-like response; and the PI3P-associated sporozoite elimination (PASE), (Figure 10B).

The most important autophagy-related process targeting *P. berghei* and similarly *P. yoelii* liver stages is the *Plasmodium*-Associated Autophagy-Related (PAAR) response. During the PAAR response LC3 is directly incorporated into the parasite’s PVM immediately after invasion. LC3 labeling of the PVM is long-lasting and occurs independent of both the ULK and PI3K complex [401–404]. New insights suggested LC3 lipidation at the PVM involves V-ATPase assembly and non-canonical recruitment of ATG16L1 (unpublished results). If this can be confirmed, the PAAR response closely resembles a process termed V-ATPase-ATG16L1-induced LC3B lipidation (VAIL)[405] and a process called Conjugation of ATG8 proteins to Single Membranes (CASM)[406]. Around 90% of *P. berghei* liver stage parasites are targeted by the PAAR response [401–403]. However, parasites provoking the PAAR response are not necessarily being eliminated, around 50% of *P. berghei* parasites persist and successfully develop (Figure 10B, PAAR response) [401]. Interestingly, infection of *FIP200^−/−^* cells that are deficient in canonical autophagy but still can execute PAAR response, resulted in a decreased parasite size. However, in comparison to wildtype cells, *FIP200^−/−^* cells could eliminate the same number of parasites suggesting that canonical autophagy is not required for parasite elimination but rather supports parasite growth by supplying additional nutrients. On the other hand, in *ATG5^−/−^* cells which are deficient in canonical autophagy and PAAR response, parasite survival is enhanced [401–403]. This suggests the PAAR response is responsible for the elimination of a significant proportion of parasites during the liver stage.

Autophagic responses of host cells vary considerably between different *Plasmodium* species investigated so far. Although some features are shared in different species, the molecular mechanisms seem to differ substantially. LC3 labeling of the PVM appears to be a common denominator during hepatocyte infection as it was observed during *P. berghei, P. yoelii* and *P. vivax* liver stage infection [401,402,407]. However, *P. vivax* parasites are targeted by a process, which rather resembles LC3-associated phagocytosis (LAP) and only targets around 30% of parasites. Similar to LAP, LC3 labeling of the PVM follows PI3P formation by the PI3K complex and leads to efficient parasite elimination (Figure 10B, LAP-like response)[407]. In contrast to the PAAR response, LC3 labeling of the *P. vivax* PVM depends on IFN-γ and does not occur spontaneously. Thus, there appears to be substantial differences in autophagic targeting between the rodent and human *Plasmodium* species. These differences might stem from their different lifestyles: *P. vivax* parasites are able to form dormant stages in the liver called hypnozoites, which can reactivate months or even years after initial infection [408]. *P. vivax* thus might have evolved a different evasion strategy that prevents immediate detection by host cell autophagy and allows dormancy. Low metabolic activity might also be a reason why at least dormant *P. vivax* parasites are not spontaneously recognized.

Interestingly, a small fraction of *P. berghei* parasites is also eliminated in a pathway resembling LAP. In the first hours of hepatocyte infection a maximum of 5 % of parasites are transiently labeled with PI3P followed by weak LC3 labeling and acidification through lysosomal fusion. This is in stark contrast to the long-lasting association of LC3 with the PVM during the PAAR response which is not preceded by PtdIns3P labeling. Surprisingly, elimination of PtdIns3P-positive parasites partly occurs also in *ATG5^−/−^* cells and is therefore not dependent on autophagy. The parasites targeted by this PtdIns3P-associated sporozoite elimination (PASE) were identified as transmigrating sporozoites which are contained within a TV (Figure 10B, PASE) [404]. To traverse cells, parasites engage perforin-like protein 1 (PLP1 also known as SPECT2) [409,410]. *In vitro* infection of hepatocytes with CT-deficient, SPECT2-negative parasites resulted in a higher percentage of parasites targeted by PASE, as they are not able to exit their TV. Sporozoites captured in their TV are successfully acidified by lysosomal fusion [404].

Although no autophagosome membrane is formed around the parasite and classical selective autophagy can be excluded as a mechanism of parasite recognition, ubiquitin and the selective autophagy receptors (SARs) SQSTM1/p62, NBR1 (neighbor of BRCA1 gene 1) and to a lesser extent NDP52 associate with the PVM of *P. berghei* parasites [401,411]. Interestingly, in *P. berghei* infected cells, labeling with SARs does not follow the selective autophagy pathway. The order of membrane labeling appears to be inverted from LC3 recruiting SARs which in turn recruit ubiquitin [411]. This raises the question, how LC3 itself is recruited to the PVM and what the function of SARs is at the PVM if not recruiting LC3.

The answer to the last question can be found in the signaling function of SQSTM1/p62. This protein is also a central hub in cell signaling as it is involved in many pathways including NF-kB signaling, nutrient sensing and NRF2-driven oxidative stress response. None of these functions depend on the LC3 interacting region (LIR) motif or the ubiquitin-binding domain that are needed for successful function of SQSTM1/p62 in selective autophagy. Therefore, SQSTM1/p62 represents a link between autophagy and signaling (reviewed in Katsuragi Y el al 2015 [412]). Interfering with host cell signaling is a popular strategy of intracellular pathogens to secure survival and establish a perfect niche for growth [413]. NRF2 is a transcription factor involved in oxidative stress response and pro-survival mechanisms. Under basal conditions, NRF2 is constantly bound by its negative regulator KEAP1, leading to ubiquitination and subsequent proteasomal degradation of the complex [414]. Sequestration of KEAP1 by SQSTM1/p62 allows stabilization and therefore activation of NRF2. Phosphorylation of SQSTM1/p62 at Ser349 drastically enhances affinity of KEAP1 for p62 (Figure 10C; bottom panel) [415]. It was recently shown that in *P. berghei*-infected hepatocytes, host cell p62 is recruited to the PVM and binds KEAP1. This KEAP1 recruitment to the *Plasmodium* PVM-associated SQSTM1/p62 appears to activate NRF2 signaling in infected hepatocytes, thereby ensuring host cell and thus parasite survival (Figure 10C; 1) [416]. Several NRF2 target genes were shown to be upregulated upon *Plasmodium* infection in a recent *in vivo single* cell transcriptomics screen. Among these were proteins involved in glutathione and iron metabolism such as glutathione S-transferases (Gstm1 and Gstm3) and Hmox1, Ftl1, Fth and Slc40a1 respectively. In addition, SQSTM1/p62 expression itself, which is also controlled by Nrf2, was found to be upregulated in infected host cells [417]. Additionally, SQSTM1/p62 is involved in other signaling pathways. It has been shown that it interacts with TRAF6 and other players in the nutrient sensing machinery of the cell [418,419]. Interaction between SQSTM1/p62 and TRAF6 is needed for activation of MTOR [419], a master regulator of nutrient sensing, promoting anabolic processes when nutrients are available and activating autophagy when nutrients are scarce [420]. Thus, SQSTM1/p62 might function as an adaptor for assembly of the nutrient sensing machinery on the lysosomal membrane and mTORC1 activation thereby regulating autophagy inhibition in response to AAs (Figure 10C; upper panel). However, it remains to be shown how the parasite interferes with the cell’s nutrient sensing and metabolic signaling to induce canonical autophagy that could provide additional nutrients for the parasite (Figure 10C; 2).

Related to the LC3 interaction to the PVM, it has been speculated whether this is beneficial or deleterious for the parasite. The answer is not straightforward as it turned out that it can be both. The PVM-resident protein UIS3 was reported to be crucial for autophagy evasion [421]. This protein interacts with LC3 and likely competes with the binding of LIR-motif containing proteins to LC3, therefore allowing evasion from autophagic degradation[421]. Under normal conditions *UIS3(-)* parasites are efficiently eliminated by the host cell as they have problems maintaining an intact PVM [422]. Importantly, *UIS3(-)* parasites can successfully develop in autophagy-deficient *ATG5^−/^*^−^, *ATG7^−/−^* or *ATG3^−/−^* cells, confirming the importance of autophagy and related pathways for parasite elimination. In the opposite side, it was shown that a small molecule, C4 (4-{[4-(4-{5-[3-(trifluoromethyl) phenyl]-1,2,4-oxadiazol-3-yl}benzyl)piperazino]carbonyl}benzonitrile), that inhibits interaction between the parasite-derived protein called Upregulated in infective sporozoites 3 (UIS3) and LC3 is capable of impairing infection [423] arguing that LC3 labeling of the PVM is beneficial for parasite survival.

Clearly, liver stage *Plasmodium* parasites need additional strategies to evade degradation by lysosomal fusion. A very efficient way to avoid autophagic responses in general is enhanced membrane shedding towards the TVN [397]. By shedding autophagy proteins and lysosomes from the PVM, the parasite evades harmful consequences of PVM targeting by the host cell. This way the parasite does not only evade elimination but might even benefit from lysosomal fusion at the TVN as an additional source of nutrients. This suggests that there is an ideal amount of lysosomal material at the PVM allowing the parasite to benefit from nutrients and lipids while avoiding lysosomal elimination. The equilibrium is maintained by constant PVM shedding. Arrested parasites that are impaired in membrane shedding exhibit a strong PVM labeling with the lysosomal marker LAMP1 followed by acidification and elimination of the parasite [424]. Interestingly, successfully developing parasites are not acidified even though lysosomes fuse with the PVM and material of the endo-lysosomal network is found in the cytoplasm of the parasite [424,425]. One possible explanation for that might be that the PVM contains pores which allow diffusion of molecules below 855 Da [395] and would thus most likely allow protons to freely diffuse into the host cell cytoplasm avoiding a drop of the pH in the PV.

In summary, autophagy and related responses can have beneficial as well as deleterious aspects for intracellular development of *Plasmodium* parasites. To benefit from the capability of providing nutrients and membrane material parasites must balance activation of autophagy and preventing elimination by autophagy-related pathways. The PVM plays a central role in both the host cell’s ability to target of the intruder and the parasite’s evasion strategy. Interestingly, a highly dynamic vesicle exchange between the host cell and the parasite appears to take place and is not only restricted to lysosomes and late endosomes but also concerns Golgi-derived vesicles [426]. The *P. berghei* PVM is able to fuse with host late endocytic vesicles in a PtdIns(3,5)P2 -dependent manner, allowing the exchange of material between the host and the parasite, which is essential for successful infection [427]. However, the precise molecular mechanism that drives the interaction between host cell vesicles and the PVM and how parasites can access the potential nutrients in the vesicles remains elusive.

Downstream molecules involved in efficient vesicle fusion are usually proteins of the SNARE family and small GTPases of the Ras-related in brain (RAB) protein family, which should be subject of future investigations. If we finally understand better the fine balance of beneficial and deleterious autophagy responses in infected hepatocytes, it might be possible to generate drugs that shift this balance to complete elimination of parasites.

### Role of autophagy on *in vivo* models of infection

#### Autophagy during trypanosomatids infection in mice

Despite the high number of *in vitro* studies showing the interplay of autophagy and parasites in different scenarios of the host cell, protist species and the types of infective forms, there is a relatively modest quantity of works describing the effect of autophagy on trypanosomatid infections in animal models. This difference is based in the increased level of complexity that means to pass from *in vitro* to *in vivo* models of infection. Similar to cell culture experiments, data obtained from mice infection have to be interpreted in the light of the type of intervention, genetic or pharmacological, used to interrupt or activate autophagy in the whole organism or in a specific tissue or organ. In the case of *T. cruzi*, first studies showed that autophagy played a protective role during the infection. Acute *T. cruzi* infection in the autophagy deficient BECN1 heterozygous knock-out mice (*Becn1^+/-^*), generated in the C57BL6/J background in the Beth Levine laboratory [428], was associated with higher parasitemia, cardiac parasitism and earlier death compared to their WT counterpart. Peritoneal cells derived from Becn1*^+/-^* animals and RAW macrophages treated with autophagy inhibitors displayed higher levels of infection compared to controls. Interestingly, in RAW cells free cytosolic parasites are surrounded by LC3 protein and other markers of selective autophagy such as Ubiquitin, SQSTM1/p62 and NDP52, features that were observed in lesser extension in autophagy-deficient cells, indicating that this process contributed to control the infection by elimination of intracellular amastigotes [353]. Strikingly, expression of inducible nitric oxide synthase (iNOS) in peritoneal cells and NO_2_^−^ plasma levels of Becn1*^+/-^* mice measured 5 days after *T. cruzi* infection showed significantly lower increase than Becn1*^+/+^*compared to uninfected animals (Casassa and Romano, unpublished data). Additionally, Duque and colleagues showed that induction of autophagy by treatment of mice with the autophagy inducer Rapamycin protected the cardiac function by reducing myocarditis, cardiac damage and the production of pro-inflammatory cytokines by the heart during acute *T. cruzi* infection without modulation of cardiac infection and parasitemia [355]. Therefore, beneficial actions of autophagy in mice infected with *T. cruzi* are displayed at different levels, from a cell-autonomous immune defense process through the engulfment of cytoplasmic amastigotes [352,353], to the modulation of inflammation and immune responses [429,430] It is known that C57BL6/J mice, that preferentially develop Th1 responses, are able to resist *T. cruzi* infection by activation of iNOS and the increase of NO level, a potent anti-*T. cruzi* agent [431]. In contrast, BALB/c mice, which are more susceptible to *T. cruzi* infection, displayed a predominance of Th2 responses and polyamine synthesis that benefit the parasite growth [432]. In this sense it was recently shown that chronic Rapamycin pretreatment contributed to improve the balance between Th1 and Th2 effectors in elderly C57BL6/J mice, which exhibited a better parasitological control, reduced heart inflammation and microstructural damage [433].

Balance between Th1 and Th2 responses is also important during the experimental infection of mice with *Leishmania amazonensis. Il4*^−/−^ BALB/c mice displayed increased Th1 response and developed smaller lesions when infected with moderate parasite inoculum compared to *Il4*^+/+^ mice. Similar results were obtained in *Il4*^+/+^ mice treated with indomethacin, an inhibitor of PGE(2) synthesis [434]. Interestingly, induction of autophagy in macrophages from BALB/c mice displayed higher parasitic load compared to macrophages from C57BL6/J mice. Increased parasite load was associated with a reduction in NO levels and increased number of lipid bodies and amounts of PGE(2) thus indicating that in macrophages from these mice strain can induce autophagy and generate products that favor *L. amazonensis* replication and reduces the production of toxic compounds at the same time [312]. Moreover in skin lesions of BALB/c mice infected with *L. amazonensis*, a positive reaction was observed by immunohistochemistry with anti-LC3 antibody indicating autophagy induction [302]. In contrast, action of autophagy in the control of *Leishmania* infection was demonstrated, as presented in the previous section, in the case of *L. major*, in which autophagy was shown to function downstream of endosomal TLRs signaling of macrophages from C57BL6/J mice [303]. In addition, rapamycin treatment reduced inflammatory lesions formed in the ears of *Leishmania*-infected C57BL/6 and *Tlr3/7/9*^−/−^ mice.

In conclusion, up to the moment the effect of autophagy on *in vivo* infections with trypanosomatids seems to be related to the type of immune response predominant in the mice strain; favoring the replication of parasites in the susceptible BALB/c mice, that preferentially develops a Th2 response, or contributing to the parasite elimination in the resistant C57BL6/J mice which preferentially activate Th1 responses.

#### Autophagy during in vivo infections of *Plasmodium*

As mentioned above autophagy inhibition by genetic manipulation impaired the liver *Plasmodium* growth mainly by the reduction of nutrients required for the fastest growing of this eukaryotic organism [399]. Therefore, it needs a constant supply of nutrients which must be provided by the host cell (reviewed in Nyboer B, et al 2018 [400]). *In vivo* experiments support these findings as in Atg5-deficient mice; parasite liver loads were significantly reduced [402]. However, the exact mechanism behind the reduced liver load is not known. While parasites in autophagy-deficient cells are clearly smaller, they can still successfully develop into detached cells harboring infective merozoites [402]. Hence, while canonical autophagy contributes to parasite growth, it is not essential for parasite development. One reason might be that the intracellular parasites also control other potential nutrient sources in that it participates in the host cell Golgi vesicular traffic [423]. However, it is not known what kind of nutrients they gain from associating with the host cell Golgi and how they are eventually processed.

Interestingly, activation of both starvation-induced and drug-mediated canonical autophagy by treatment with the MTOR inhibitor rapamycin results in an extraordinary 25-fold increase in parasite load *in vivo*, that arises from significantly increased parasite size but mainly from enhanced parasite survival [401]. The mechanism underlying enhanced parasite survival upon activation of canonical autophagy induction is still unknown, but it is remarkable that the *in vivo* effect of autophagy activation is much stronger than in *in vitro* cultivated cells including primary hepatocytes. A reason could be that *in vivo*, autophagy responses are more pronounced because they underlie physiological conditions, whereas *in vitro*, medium conditions including supplements and fetal calf serum downregulate such autophagy responses. It is also important to note that increased growth of parasites in rapamycin-treated mice could be due to the immunosuppressive effect of this compound *in vivo* [435,436] resulting in the impairment of the immunity against *Plasmodium*. An interesting hypothesis concerning the extraordinary growth and survival of liver stage parasites *in vivo* upon rapamycin treatment or starvation is that different autophagy pathways might compete for limited concentration of autophagy proteins. In normal cells most autophagy-related proteins (i.e. LC3) are free in the cytosol. Upon parasite invasion, autophagy proteins are sufficient to target the parasite and about 50% are eliminated by the PAAR response. In contrast, when canonical autophagy is induced by starvation or rapamycin treatment, autophagy-related proteins are required for the generation of autophagic vesicles and less parasites are attacked by the PAAR response. Together, the autophagic status of the host cell appears to drastically influence the outcome of infection *in vivo* and to a lesser extent also *in vitro*. The beneficial effect of autophagy can be modulated by the same parasite. It is showed that *Plasmodium yoelii* rodent malaria parasites perturb hepatocyte regulatory pathways involved in cell survival, proliferation, and autophagy. The pro-death protein p53 was substantially decreased in infected hepatocytes suggesting that it could be targeted by the parasite to foster survival [437]. In contrast, other host pathways are hijacked by *Plasmodium* as was shown by the detrimental parasite infection observed during the knockdown of genes involved in protein trafficking pathways [438]. Targeting of host proteins might be a smart strategy to expand the repertoire of prophylactic drugs against malaria as in the case of p53 agonists that were effective when administered to humanized mice infected with *Plasmodium falciparum* [439].

#### Effect of host autophagy on *T.* *gondii* infection *in* *vivo*

As discussed above, the most important mechanism of *T. gondii* to avoid killing by the host cell is the formation of a specialized intracellular compartment (the PV) that does not fuse with compartments from the endolysosomal system. The two main host mechanisms to induce parasite killing, and control toxoplasmosis, involving either autophagy or autophagy proteins, have been recently reviewed focusing on the importance of these processes in the outcome of *in vivo* infections [440]. The autophagy-dependent killing of parasites is activated by CD40 and its main ligand CD154 [361]. Existence of this mechanism was confirmed *in vivo* in CD40^–/–^ and CD154^–/–^ mice, which displayed increased susceptibility to cerebral and ocular toxoplasmosis [373,441] despite seemingly unimpaired expression of IFN-γ, the main cytokine that mediates protection against *T. gondii* in mice [442,443]. Similar increased susceptibility to cerebral and ocular toxoplasmosis was demonstrated in Becn1^+/-^ mice, mice lacking Atg7 in myeloid cells (Atg7flox/flox-Lyz-M Cre mice), and mice deficient in the pro-autophagy protein PKR (PKR^–/–^) [373,444]. This confirms that CD40-induced death of *T. gondii* (type I strain) is independent of IFN-γ and effector responses downstream of this cytokine and depends on autophagy. Besides macrophages and microglia, CD40 restriction of infection is also functional in brain endothelial cells, an important port of entry of *T. gondii* to the Central Nervous System (CNS), mainly the brain and retina [445]. The toxoplasmicidal actions of endothelial cells is acquired upon interaction of these cells with *T. gondii*-infected dendritic cells and macrophages, and depends on the expression of CD40 by endothelial cells and the expression of BECN1 and the inducible heat shock protein 70 in dendritic cells [445]. Other actions of CD40 pathway include reduced anti-*T. gondii* antibody production [446] and protection against CD8+ T cell exhaustion[447], which have implications in the establishment of the chronic infection.

The second mechanism to control *T. gondii* infection is displayed by cell-autonomous actions elicited by INF-γ in which autophagy proteins are involved. In this case, parasite killing is independent of endosomal/lysosomal fusion with the PV and depends on the recruitment of certain autophagy proteins Atg3, Atg7, and the complex Atg5-Atg12-Atg16L [366,448]. In mouse cells these proteins trigger the recruitment of GKS IRGs to the PVM, followed by deposition of ubiquitin, SQSTM1/p62, and the E3 ubiquitin ligases TRAF6 and TRIM21, which promotes sustained decoration of PVM with ubiquitin [449]. This process leads to delivery of guanylate-binding proteins (GBPs) to PVM followed by vesiculation and rupture of the vacuole, and finally death of susceptible strains [448,450], type II and III (Figure 9B, left panel). Virulent *T. gondii* strains (type I) avoid this action by the production of ROP kinases that acts synergistically to impair the recruitment of IRGs to the PVM, and control acute infection in mice [451]. Control of *T. gondii* infection by INF-γ in human cells is quite different than in mouse cells since it does not rely on IRGs and has notable cell-type specificity. In human epithelial cells infected with type II and III strains, ubiquitin deposition on PVM leads to recruitment of SQSTM1/p62, NDP52, and optineurin receptors followed by the formation of a LC3-positve multilayer that restricts the parasite replication [370] (Figure 9B, central panel). In contrast, in human endothelial cells infected with type-II strain, ubiquitin and SQSTM1/p62 deposition is followed by recruitment of Rab7, fusion with lysosomes, and parasite killing [452] (Figure 9B, right panel). In this case LC3 is not recruited around PVM, and this process is not dependent on autophagy [452].

IFN-γ-mediated immunity against *T. gondii* seems to be more critical in mice than humans. Patients with an autosomal dominant defect in IFN-γR1 resulting in deletion of the signal transducer and activator of transcription 1 (STAT1) binding site are not susceptible to toxoplasmosis, although their macrophages are unable to display anti-*T. gondii* activity in response to IFN-γ [453]. Treatment with tumor necrosis factor-alpha (TNF-α) partially restored antimicrobial activity of macrophages from these patients, indicating that this factor contributes to the control of infection [453]. CD40 may also contribute to IFN-γ-independent mechanisms of protection in humans [454], likely in part by inducing TNF-α production [455,456].

To investigate *T. gondii* evasion of host autophagic clearance *in vivo*, the role of EGF and EGFR was demonstrated in transgenic mice that express a dominant-negative (DN) mutant of EGFR that impairs EGFR autophosphorylation and EGFR transactivation. Expression of DN EGFR exclusively in endothelial cells was accompanied by a reduction in parasite load and histopathology in the brain and retina after *T. gondii* infection [457].

In summary, the main host mechanisms that operate *in vitro* against *T. gondii* have been investigated in mouse models of infection. Remarkably, in contrast to toxoplasmicidal actions triggered by INF-γ that are not operative across species, parasite strains, or host cell types, autophagic killing functions in all scenarios. This feature points to autophagy modulation (or impairment of *T. gondii* evasion of autophagy) as a potential strategy for drug development against toxoplasmosis.

#### Effect of parasite autophagy on apicomplexan infections *in* *vivo*

Few *in vivo* infection studies have been performed on parasite autophagy in apicomplexans for two main reasons. First, much of the work on autophagy in malaria parasites has been done with *P. falciparum*, for which animal models are limited. Second, as noted above, many ATG proteins in apicomplexans are essential for parasite replication because of their non-canonical role in apicoplast inheritance. Since conditional disruption of ATG proteins involved in apicoplast inheritance severely affects parasite replication, the impact on *in vivo* infection is assumed to be equally pronounced.

As noted above, overexpressing ATG8 by 2-fold significantly delayed the liver stage development of rodent infective *P. berghei* parasites in infected mice [175], likely due to disrupting both the canonical and non-canonical roles of ATG8.

In the case of *T. gondii*, two studies have examined the canonical role of the parasite ATG9 in autophagy during experimental infection of mice [180,458]. The first study showed that complete ablation or conditional knockdown of TgATG9 markedly reduced parasite burden during acute infection and significantly attenuated parasite virulence, resulting in >70% mouse survival with the knockout strain and or 100% mouse survival from the conditional knockdown [180]. This is particularly notable given that the mutants were created in a highly virulent strain of *T. gondii* that normally causes 100% mortality. Disruption of TgATG9 also reduced parasite levels in mouse macrophages. Since macrophages are known to restrict *T. gondii* by limiting certain essential amino acids and by generating reactive oxygen species, the authors suggested that parasite autophagy supports parasite survival by recycling amino acids or turning over oxidatively damaged parasite proteins, structures, or organelles. The second study knocked out TgATG9 in an avirulent *T. gondii* strain that is competent for differentiating from the acute to chronic stage [458]. Consistent with the earlier study, mice infected with parasites lacking TgATG9 showed 100% survival compared to 70-80% survival of mice infected with parental or genetically complemented strains. Also, ~5-fold fewer TgATG9 parasites were detected upon dissemination to the brain on day 7 post-infection, further supporting a role for canonical autophagy during acute infection. However, TgATG9 deficient parasites showed a >200-fold loss of parasite burden in the brain at 5 weeks post-infection during the chronic stage. Death of chronic stage TgATG9 deficient parasites *in vitro* was associated with markedly abnormal mitochondria, implying a critical role for autophagy in support of mitochondrial homeostasis. Whether the canonical autophagy pathway in *T. gondii* has additional roles during persistence warrants further investigation.

## Concluding remarks

In this work, we have reviewed the biological cycle of pathogenic protists and the participation of the autophagic pathway in the different stages of their cycles. The particular lifestyles of these parasites, which alternate between vertebrate and invertebrate hosts, in addition to the necessity to adapt to different environments, allow the conservation of cellular processes like autophagy. We have seen that autophagy displayed different levels of complexity through this early evolutionary branch of the eukaryote domain, from a rudimentary process observed in the *Giardia* genus up to the more complex pathway displayed in Trypanosomatids and Apicomplexa families with the diversification of autophagic proteins such as Atg4 and Atg8 in a similar way to what is observed in humans, a feature whose presence is still not completely understood. Parasite autophagy has been shown to participate in many processes, including survival under nutrient deprivation, formation and turnover of organelles, and cell and nuclear death during specific situations. Contrary to these physiological functions, autophagy is activated in the presence of antiprotist drugs as a quick survival response to the stress generated by the treatment. Since this is a phenomenon observed with most drugs, the use of inhibitors of parasite autophagy as adjuvants of anti-parasitic drugs could be interesting to explore in the future.

The interaction of pathogenic parasites with the host autophagy has also been reviewed. In most cases, autophagy is activated in the host cell after infection, being a protective response for the host, which can be subverted by the pathogen to favor growth and survival. Therefore, depending on parameters like type of the host cell-targeted, time of infection, and life-styles and virulence of pathogenic protists, host autophagy plays two opposite roles, promoting parasite survival or parasite death. More complex is the scenario when the function of autophagy is studied on *in vivo* models of infection, where the immune system can modulate the autophagy response and vice versa, modifying the fate of infection. Finally, we have to consider that parasitic autophagy can also play an important role in the intracellular protist life, another aspect that should be considered for future treatments. In summary, protist autophagy is needed at different stages of the protist life-cycle, in axenic environments, or inside host cells. On the other way, host cells have developed different mechanisms to destroy parasites during infection. They are carried out by canonical or selective autophagy pathways, autophagy proteins, and/or autophagy-related processes to finally kill the pathogen. However, virulent pathogens can take advantage of host autophagy responses to resist destruction. The application of this knowledge in the search and design of new drugs that interrupt the mechanisms of resistance of protists in addition to the improvement of immune responses and cell-autonomous processes of hosts will allow the discovery of efficient therapies against these still successful pathogens in the future.
